# Interspecies and Intraspecies Analysis of Trehalose Contents and the Biosynthesis Pathway Gene Family Reveals Crucial Roles of Trehalose in Osmotic-Stress Tolerance in Cassava

**DOI:** 10.3390/ijms17071077

**Published:** 2016-07-13

**Authors:** Bingying Han, Lili Fu, Dan Zhang, Xiuquan He, Qiang Chen, Ming Peng, Jiaming Zhang

**Affiliations:** Institute of Tropical Bioscience and Biotechnology, Key Laboratory of Tropical Crops Biology and Genetic Resources, Ministry of Agriculture, Hainan Bioenergy Center, Chinese Academy of Tropical Agricultural Sciences (CATAS), Xueyuan Road 4, Haikou 571101, China; hanbingying@itbb.org.cn (B.H.); fulili@itbb.org.cn (L.F.); 18777350551@163.com (D.Z.); xiuquan.he@163.com (X.H.); 13666906069@163.com (Q.C.); pengming@itbb.org.cn (M.P.)

**Keywords:** *Manihot esculenta*, drought tolerance, HPLC-ELSD, phylogenetic analysis, trehalase, abiotic stress, RNA-seq

## Abstract

Trehalose is a nonreducing α,α-1,1-disaccharide in a wide range of organisms, and has diverse biological functions that range from serving as an energy source to acting as a protective/signal sugar. However, significant amounts of trehalose have rarely been detected in higher plants, and the function of trehalose in the drought-tolerant crop cassava (*Manihot esculenta* Crantz) is unclear. We measured soluble sugar concentrations of nine plant species with differing levels of drought tolerance and 41 cassava varieties using high-performance liquid chromatography with evaporative light-scattering detector (HPLC-ELSD). Significantly high amounts of trehalose were identified in drought-tolerant crops cassava, *Jatropha curcas*, and castor bean (*Ricinus communis*). All cassava varieties tested contained high amounts of trehalose, although their concentrations varied from 0.23 to 1.29 mg·g^−1^ fresh weight (FW), and the trehalose level was highly correlated with dehydration stress tolerance of detached leaves of the varieties. Moreover, the trehalose concentrations in cassava leaves increased 2.3–5.5 folds in response to osmotic stress simulated by 20% PEG 6000. Through database mining, 24 trehalose pathway genes, including 12 trehalose-6-phosphate synthases (TPS), 10 trehalose-6-phosphate phosphatases (TPP), and two trehalases were identified in cassava. Phylogenetic analysis indicated that there were four cassava *TPS* genes (*MeTPS1–4*) that were orthologous to the solely active *TPS* gene (*AtTPS1* and *OsTPS1*) in *Arabidopsis* and rice, and a new TPP subfamily was identified in cassava, suggesting that the trehalose biosynthesis activities in cassava had potentially been enhanced in evolutionary history. RNA-seq analysis indicated that *MeTPS1* was expressed at constitutionally high level before and after osmotic stress, while other trehalose pathway genes were either up-regulated or down-regulated, which may explain why cassava accumulated high level of trehalose under normal conditions. *MeTPS1* was then transformed into tobacco (*Nicotiana benthamiana*). Results indicated that transgenic tobacco lines accumulated significant level of trehalose and possessed improved drought stress tolerance. In conclusion, cassava accumulated significantly high amount of trehalose under normal conditions due to multiplied trehalose biosynthesis gene families and constant expression of the active *MeTPS1* gene. High levels of trehalose subsequently contributed to high drought stress tolerance.

## 1. Introduction

Cassava (*Manihot esculenta* Crantz) is a perennial dicotyledonous crop belonging to the family of Euphorbiaceae. The starchy tuberous roots have global importance as food and feed, and are stable food for nearly 700 million people in the tropical and subtropical regions [1,2]. The starches in cassava roots have high value as precursors for industrial products [1,2]. Small-scale farmers contributed more than 70% of the cassava roots, with a total cultivated area of more than 18 million hectares [2]. Cassava is highly tolerant to abiotic stress and can be grown on arid and marginal land [3,4,5]. The mechanisms for this tolerance have been studied on physiological and transcriptional aspects [3,6], but still remain obscure.

Trehalose is a nonreducing α-d-glucopyranoside disaccharide. It is composed of two d-glucose molecules linked with an α,α-1,1-glycosidic bond, resistant to acid hydrolysis and high temperature, and is generally inert in its interactions with proteins [7]. Trehalose was first identified by Wiggers in 1832 in ergot-infected rye and its properties and history was well-reviewed [7,8]. Trehalose has been detected in a wide range of organisms and possesses many biological functions ranging from serving as an energy source to acting as a protective and/or signal sugar against abiotic stress [9,10,11,12,13]. Trehalose is very common in yeast and fungi [14,15]. The spores and macrocysts of *Dictyostelium mucoroides* was reported to contain 7% trehalose on a dry-weight basis [16], while the ascospores of *Neurospora tetrasperma* was reported to contain 10% trehalose [17]. The accumulated trehalose disappeared rapidly upon spore germination suggesting a storage role for trehalose [16,17]. Yeast was also reported to accumulate trehalose in high concentration during nutrient limitation [18] and under environmental stress [19].

Some resurrection plants are able to accumulate trehalose in concentrations as high as 36 mg·g^−1^ dry weight (DW) under dehydration condition [20,21]. These plant species can tolerate and survive extreme vegetative desiccation, and resume normal cellular metabolism within a short period after re-watering [21,22,23]. However, in the majority of higher plants, trehalose is present in trace or undetectable amounts, but the detected trace amount was not repeatable and was once suspected to be microbial contamination [24]. Endogenous trehalose has been detected in the model plant *Arabidopsis thaliana* in trace amount by using validamycin A, a trehalase inhibitor in the culture medium [25]. A later research reported a level of 0.01 mg·g^−1^ fresh weight (FW) of trehalose in seven-day-old plantlets of wild type *Arabidopsis* [26]. Trehalose has also been detected in wild type rice (*Oryza sativa*) with a level of approximately 0.015 mg·g^−1^ FW [27] and wild type tobacco (*Nicotiana tabacum*) with a level of 0.001 to 0.002 mg·g^−1^ FW [28]. Because of the low concentrations or absence of trehalose in a vast majority of higher plants, its biological function in higher plants remained a mystery for a long time [29,30]. However, multiple biological functions of trehalose have been proposed in higher plants recently, including membrane and protein stabilization [31], and the regulation of plant growth and development, particularly during embryo maturation, the transition from vegetative growth to flowering, inflorescence architecture, seedling development, and in stress signaling [32,33,34].

In this paper, we report unexpected high levels of trehalose in all tested cassava varieties, which is highly correlated with dehydration stress, and phylogenetic and transcriptional analysis of trehalose biosynthesis gene family. The high level accumulation of trehalose in cassava may be explained by multiplied trehalose-6-phosphate synthase (*TPS*) and trehalose-6-phosphate phosphatase (*TPP*) gene families and constant expression of a key active *TPS* gene.

## 2. Results

### 2.1. Cassava Has High Level of Trehalose under Normal Conditions

**Figure 1:**
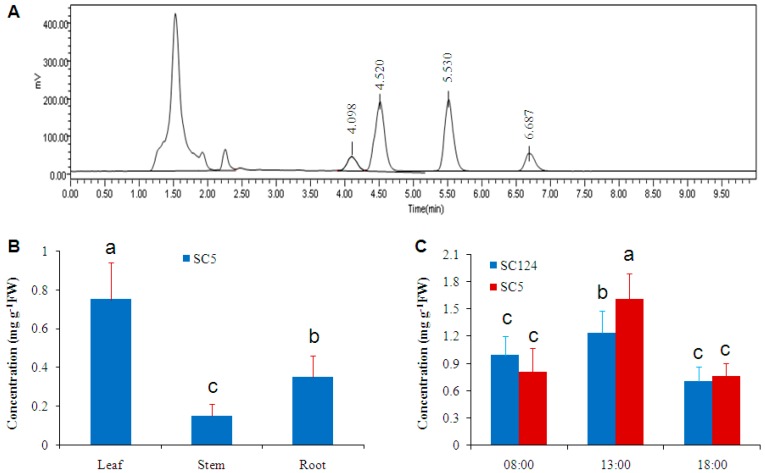
Trehalose concentrations in cassava varieties SC5 and SC124: (**A**) HPLC chromatograph of soluble sugars in cassava (SC5) leaf; (**B**) distribution of trehalose in cassava organs (SC5); and (**C**) circadian oscillations of trehalose contents in leaves of cassava varieties SC124 and SC5. The significance of differences was tested by one-way ANOVA and LSD (Least Significant Difference) test using IBM SPSS Statistics Version 24.0 (IBM Corporation, New York, NY, USA). Different letters above columns indicate significant differences at 1% level of significance.

High-performance liquid chromatography with evaporative light-scattering detector (HPLC-ELSD) was used to measure soluble sugar contents in cassava. Preliminary results showed that soluble sugars were well separated using our protocols and parameters, with retention times of approximately 4.098, 4.520, 5.530, and 6.687 min for fructose, glucose, sucrose, and trehalose, respectively, in leaf samples (Figure 1A). Subsequently, this method was applied to measure soluble sugar contents in different tissues of cassava (variety SC5), and high level of trehalose was revealed (Figure 1B): leaves accumulated the highest levels, followed by root and stem.

Trehalose level in cassava varied with respect to a circadian cycle under normal conditions (Figure 1C). At 8:00 a.m., trehalose contents in the leaves of varieties SC124 and SC5 were 0.99 and 0.81 mg·g^−1^ FW, respectively. At 1:00 p.m., trehalose contents reached the highest level. At 6:00 p.m., trehalose contents declined to similar level as in the morning (Figure 1C).

### 2.2. Trehalose Level Is Species-Dependent in Crops

**Figure 2:**
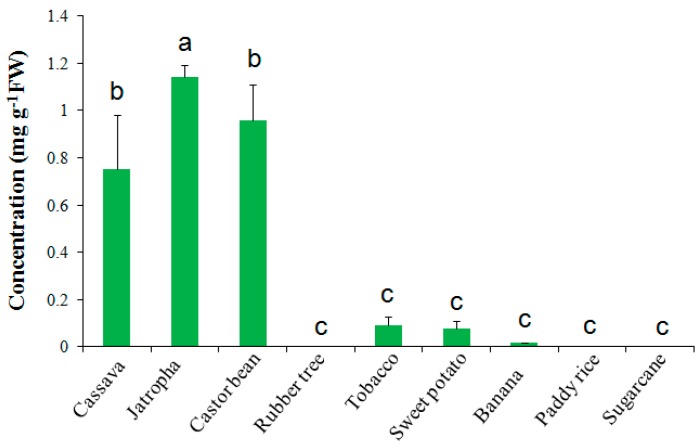
Trehalose concentrations in leaves of different plant species under normal conditions. The contents were measured with HPLC-ELSD method, and presented in mg·g^−1^ FW (Fresh Weight). The significance of differences was tested by one-way ANOVA, followed by LSD test. Different letters above columns indicate significant differences at 1% level of significance, as analyzed using IBM SPSS Statistics Version 24.0.

To investigate whether other crops also contained high level of trehalose, leaves of several crops that are cultivated in South China, including *Jatropha curcas*, rubber tree, castor bean, sweet potato, rice, banana, and sugarcane, together with a laboratory plant tobacco, were examined. Interestingly, *Jatropha* and castor bean leaves had high trehalose contents of 1.14 and 0.96 mg·g^−1^ FW, respectively (Figure 2). These two species, together with cassava, all belong to Euphorbiaceae family, and are considered as drought-tolerant plants compared to other species shown in Figure 2, since they can be grown in arid and marginal lands [4,5,35,36]. Rubber tree is another Euphorbiaceae family member; however, it did not accumulate detectable levels of trehalose (Figure 2). Trehalose contents in sugarcane and rice leaves were also below the level of detection. Trace amount of trehalose (0.02–0.09 mg·g^−1^ FW) was detected in banana, tobacco, and sweet potato leaves (Figure 2).

### 2.3. Trehalose Level Is Genotype-Dependent in Cassava and Positively Correlated with Dehydration Tolerance

**Figure 3:**
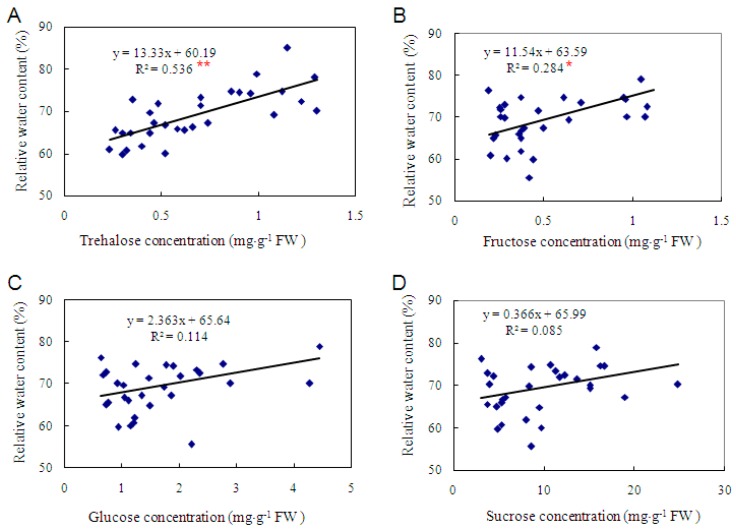
Correlations between soluble sugar contents including: trehalose (**A**); fructose (**B**); glucose (**C**); and sucrose (**D**) in different cassava varieties and water retaining capacities of their detached leaves. The significance of the correlations was analyzed using IBM SPSS Statistics Version 24.0, and the correlation curves were created with Excel 2007 (Microsoft Inc., Seattle, WA, USA). ** and * indicate significant correlations at 1% and 5% levels of significance, respectively.

To investigate the biological function of trehalose in cassava, a variety with low trehalose level is desired. However, all 39 cassava varieties that were randomly selected from the cassava germplasm bank of CATAS (Chinese Academy of Tropical Agricultural Sciences) contained similar high levels of trehalose as varieties SC5 and SC124 (Figure 1), although the levels varied from 0.23 to 1.3 mg·g^−1^ FW (Figure 3A). Regression analysis revealed a significant correlation coefficient (*R^2^* = 0.536, *p* < 0.01) between trehalose concentration and water retaining capacity of detached leaves (Figure 3A). The results suggested that cassava leaves with higher trehalose concentrations lose water more slowly than the leaves with lower trehalose.

Fructose, glucose, and sucrose were also detected in all varieties, and sucrose was the most abundant soluble sugar, with concentrations ranging from 2.22 to 16.23 mg·g^−1^ FW (Figure 3). The concentrations of these potential osmolytes were also positively correlated with water retaining capacity, but the correlation coefficients were not significant except for fructose, which was 0.284 (*p* < 0.05) (Figure 3B–D). These results suggested that trehalose was potentially more important in osmotic stress tolerance in cassava than fructose, glucose, and sucrose.

### 2.4. Osmotic-Stress Stimulates Trehalose Accumulation in Cassava

**Figure 4:**
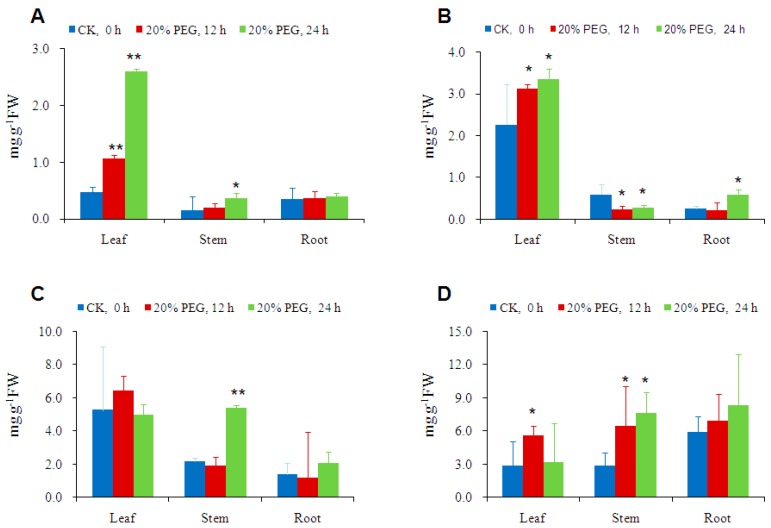
Changes of soluble sugar concentrations in different tissues of cassava (SC5) in response to 20% PEG treatment for 12 and 24 h: (**A**) Trehalose; (**B**) Fructose; (**C**) Glucose; and (**D**) Sucrose. The significance of differences were assayed by one-way ANOVA and LSD test using IBM SPSS Statistics Version 24.0. * and ** above columns indicate significant differences compared to Control (CK, 0 h of PEG treatment) under 5% and 1% levels of significance, respectively.

To investigate the response of trehalose level to osmotic stress, cassava plants were treated with 20% PEG 6000 solution in the pots. Trehalose levels in leaves displayed the most rapid and significant increase, which were 2.3 and 5.5 folds of the control level, respectively, in 12 and 24 h of treatments. Trehalose contents in stems increased 2.5 folds in 24 h. However, trehalose contents in root remained almost unchanged (Figure 4A). The other soluble sugars including fructose, glucose, and sucrose did not show significant and consistent changes within 24 h of osmotic stress. Fructose content was predominantly present in leaves compared to stem and root, and slightly increased upon PEG treatment (statistically not significant), but decreased in stem and root in 12 h of stress (Figure 4B). Glucose content was almost unchanged in leaves and roots, but increased significantly after 24 h PEG treatment in stem (Figure 4C). The changes of sucrose contents in leaf, stem, and root were not significant due to high deviations (Figure 4D). These results further proved that trehalose is a more active and consistent player in osmotic stress response than the other soluble sugars in cassava.

### 2.5. Classification of Cassava Trehalose Pathway Enzymes

To understand the trehalose-accumulating mechanism in cassava, we performed genome-wide analysis, and identified 24 putative trehalose pathway genes, including 12 putative trehalose-6-phosphate synthase (*TPS*) genes, ten putative trehalose-6-phosphate phosphatase (*TPP*) genes, and two trehalase (*TRE*) genes from the cassava genome databases (Table S1). All these genes encoded full length enzymes, except *MeTPP6*, which lacked a few residues on the carboxyl-terminal due to incomplete sequencing; however, all motifs required for TPS and TPP activity were present. Some small *TPS* and *TPP* gene fragments were also identified in the cassava genome, but they were unlikely to encode functional enzymes, and were not investigated further.

**Figure 5:**
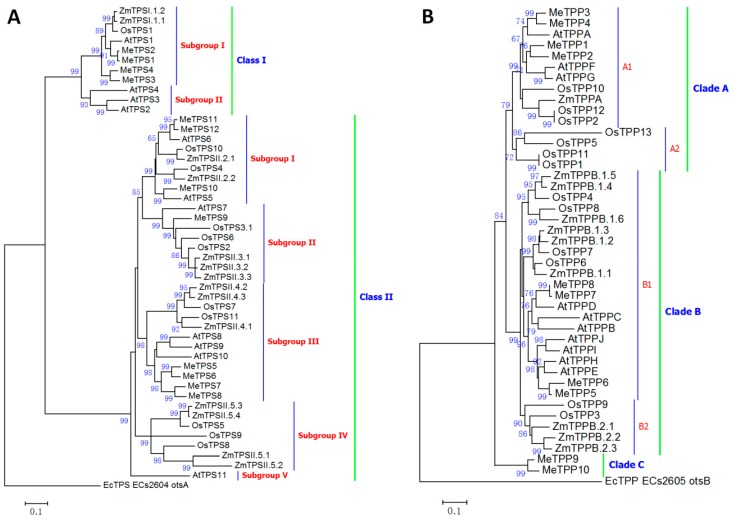
Phylogenetic classification of trehalose-6-phosphate synthase (TPS, **A**) and trehalose-6-phosphate phosphatase (TPP, **B**) proteins. The sequences were aligned with ClustlX2 [41], and phylogenies were built using MEGA 7 [42]. Maximum Likelihood (ML), Neighbor-Joining, and Minimum-Evolution methods were used in the phylogenetic analysis and only the ML trees are shown. The confidence probabilities (multiplied by 100) that the interior branch length is greater than 0, as estimated using the bootstrap test (1000 replicates), are presented at the relevant nodes. Scale bars represent 0.1 residue substitution.

Cassava TPS proteins were classified into two clades: Class I and Class II, which were subdivided into two and five subgroups, respectively, similar to the TPS family of maize, rice, and *Arabidopsis* (Figure 5A) [37,38,39,40]. Four cassava TPS proteins (MeTPS1–4) were classified into Subgroup I of Class I, together with AtTPS1 from *Arabidopsis* and OsTPS1 from rice, no cassava TPS proteins were grouped with the other three Class I *Arabidopsis* TPS proteins in Subgroup II (Figure 5A). AtTPS1 and OsTPS1 have been demonstrated to be the only active TPS proteins in *Arabidopsis* and rice, respectively, and all others lack TPS activity [38,39]. Therefore, cassava may have four active TPS enzymes. Eight cassava TPS-like proteins (MeTPS5–12) were classified into Class II, together with seven, ten, and 12 TPS-like proteins in *Arabidopsis*, rice, and maize, respectively. All Class II TPS-like proteins in *Arabidopsis* and rice have been shown to be inactive [38,39]. Therefore, it is likely that Class II TPS-like proteins in cassava are also inactive, although they all possessed full TPS and TPP domains and the motifs required for TPS and TPP activities (Figure S1). However, all Class I proteins lacked the first motif required for the catalytic activity of TPP in the phosphatase domain, similar to the Clade A or Class I TPS in maize, rice, and *Arabidopsis* [37,38,39,40].

TPP in *Arabidopsis*, rice, and maize were classified into two clades (Figure 5B). However, cassava TPP proteins were classified into three clades (Figure 5B). Four (MeTPP1–4) were located in Clade A, and four in Clade B. Interestingly, the other two, MeTPP9 and MeTPP10, were not grouped with any TPP proteins from *Arabidopsis*, rice, and maize, and formed an independent clade, designated as Clade C. All cassava TPP proteins possessed three conserved motifs required for TPP activity (Figure S2), especially for the first motif: DXDX(T/V)(L/V/I) [43,44], where a single form DYDGTL was used.

Totally, three trehalase (TRE) genes (cassava4.1_004491m.g, cassava4.1_023202m.g, and cassava4.1_015916m.g) were identified. Cassava4.1_004491m.g encoded a full-length CDS and was designated as *MeTRE1*, while cassava4.1_023202m.g and cassava4.1_015916m.g encoded the 5′ and 3′ sequences of a *TRE*, respectively. Through 5′ RACE of cassava4.1_015916m.g and sequence comparison, cassava4.1_023202m.g was revealed to be identical to the 5′ end of cassava4.1_015916m.g. Therefore, they were designated as *MeTRE2*.

### 2.6. Differential Expression Analysis of Trehalose Pathway Genes Using RNA-Seq Data

**Figure 6:**
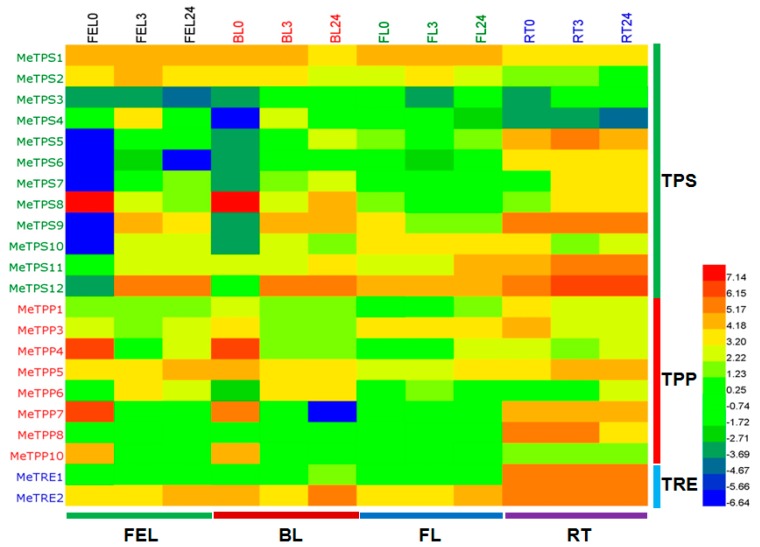
Differential expression of trehalose pathway genes in leaves and roots of cassava in response to 20% PEG treatment. These data came from a previous RNA-seq analysis [6]. The expression levels of trehalose pathway genes in all samples in TPM (Transcripts Per kilobase Million) were compiled with Excel 2007, and normalized using logarithmic method with two as base. The normalized dataset was then exported to HemI toolkit [45], and the figure (Heatmap) was built using the default parameters. The expression levels are represented by different colors as indicated by the scale on the bottom right. FEL, full expanded leaf; BL, bottom leaf; FL, folded leaf; RT, root. FEL0, FEL3, and FEL24 indicate full expanded leaf after 0, 3, and 24 h after PEG treatment, respectively; and the treatment lengths of BL, FL, and RT samples were indicated the same as above.

The transcriptome profiles of cassava leaves and root before and after treatment with 20% PEG 6000 [6] were used to study the expression levels of the trehalose pathway genes. All the *TPS*, *TPP*, and *TRE* genes were transcribed in cassava leaves and root, except for *MeTPP2* and *MeTPP9*, which were absent in the transcriptomes of leaves and root (Figure 6).

*MeTPS8*, *MeTPS2*, and *MeTPS1* (listed according to expression levels from high to low) were the major *TPS* genes expressed in the control samples of leaves including full expanded leaf (FEL) that was active in photosynthesis, bottom leaf (BL) that was at the bottom of canopy and usually aged, and folded leaf (FL) that was newly emerged and not expanded, while *MeTPS9*, *MeTPS12*, *MeTPS11*, and *MeTPS5* were highly expressed in the control samples of root (RT). The expression level of *MeTPS1* was almost constant and high before and after treatment with 20% PEG, suggesting that *MeTPS1* served as a housekeeping TPS enzyme and may explain the high trehalose level in cassava under normal conditions. The other supposed active TPS genes *MeTPS2* and *MeTPS4* had lower expression levels under normal conditions compared to *MeTPS1*, but were up-regulated in functional leaf after treated with PEG for 3 h. Interestingly, *MeTPS8*, the most abundant *TPS* in FEL0 and BL0 (Figure 6), was down-regulated in FEL3 and BL3 (3 h after PEG treatment). In root, six *TPS* genes were up-regulated after PEG treatment, while three *TPS* genes remained unchanged, and other three were down-regulated.

Eight out of ten *TPP* genes were transcribed in leaves and root of cassava. *MeTPP4*, *MeTPP7*, and *MeTPP10* were the major *TPP* genes transcribed in FEL0 and BL0 (Figure 6). However, they were all down-regulated after PEG treatment. *MeTPP5* and *MeTPP6* that were expressed at relatively lower levels than *MeTPP4*, *MeTPP7*, and *MeTPP10* under normal conditions were up-regulated in FEL after PEG treatment (Figure 6). In contrast, the expression levels of most TPP family proteins in FL were lower than those in FEL and BL (Figure 6), and *MeTPP3* was the major TPP in FL, and its expression level in FL was almost constant before and after PEG treatment, while the less highly expressed *MeTPP5* and *MeTPP6* were up-regulated after PEG treatment. In root, *MeTPP8*, *MeTPP7*, *MeTPP3*, and *MeTPP5* were the major TPPs under normal conditions. *MeTPP8* and *MeTPP3* were down-regulated upon PEG-treatment, while *MeTPP5* was up-regulated, and *MeTPP7* remained unchanged.

Trehalase (TRE) expression was most abundant in root compared to leaves (FEL, BL, and FL), and the two trehalase genes *MeTRE1* and *MeTRE2* were almost equally expressed and remained constant upon PEG treatment in root. In leaves (FEL, BL, FL), the expression level of *MeTRE2* was dominant over *MeTRE1*, and it was up-regulated in FEL24 and FL24. The changes of the expression levels of *MeTRE1* in leaves were small (Figure 6).

### 2.7. Verification of Trehalose-6-P Synthase Activity of MeTPS1

**Figure 7:**
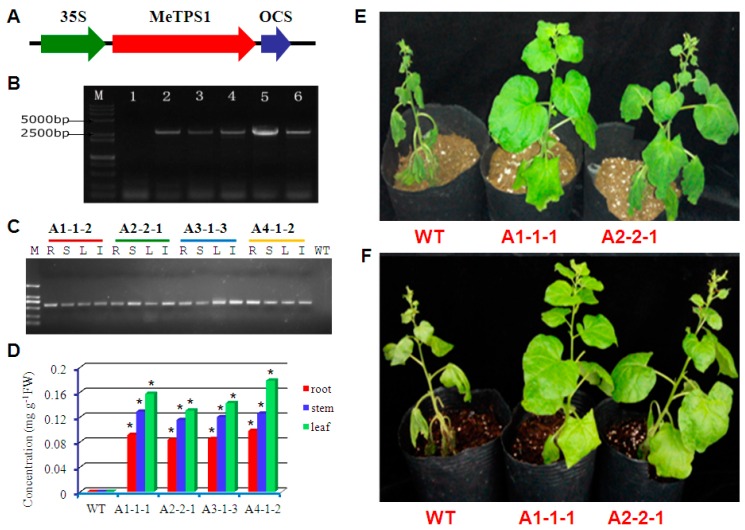
Functional analysis of *MeTPS1* gene by transforming it into tobacco: (**A**) *MeTPS1* gene was inserted in the plant expression vector *pCAMBIA2300* under the control of *CaMV35S* promoter; (**B**) PCR verification of transgenic tobacco plants (*Nicotiana benthamiana*) (Lanes: M, molecular weight marker; 1, wild type tobacco; 2, A1; 3, A2; 4, A3; 5, A4; 6, *pCAMBIA2300*-*MeTPS1*); (**C**) RT-PCR analysis of *MeTPS1* gene in homozygous transgenic lines (Lanes: M, molecular marker; R, root; S, stem; L, leaf; I, inflorescence; WT, wild-type control); (**D**) trehalose concentrations in homozygous transgenic lines, where * indicates significant differences at 1% level of significance compared to the relevant organ of wild type (WT); (**E**) representative transgenic plants and wild-type (WT) after 30 days of water-withheld; and (**F**) the same plants as in (**E**) one day after re-watering.

*MeTPS1* seemed to be the most important *TPS* in cassava according to the phylogenetic and RNA-seq data. However, MeTPS1, unlike other Class I TPS (AtTPS1, OsTPS1, ZmTPS1.1.1, and MeTPS2–4), used an aspartic acid (D121) instead of a conserved glycine residue as the first binding site to the ribose moiety in UDPG (UR1, Figure S1). To test whether this enzyme is active, plant expression vector of *MeTPS1* gene (pCAMBIA2300-*MeTPS1*) was constructed under the control of the CaMV 35S promoter (Figure 7A), and was transformed into tobacco (*Nicotiana benthamiana* Domin) using *Agrobacterium tumefaciens* GV3103. The presence of *MeTPS1* in tobacco genome was confirmed by PCR (Figure 7B). Four homozygous transgenic lines (A1-1-1, A2-2-1, A3-1-3, and A4-1-2) were obtained after selection on hygromycin containing medium for two generations. RT-PCR showed that *MeTPS1* gene was expressed in leaf, stem, root, and inflorescence of transgenic tobacco (Figure 7C). HPLC analysis revealed that significant amounts of trehalose were accumulated in leaf, stem, and root of the transgenic lines, while the wild-type control did not contain a detectable amount of trehalose (Figure 7D). The transgenic lines showed increased tolerance to natural drought, and showed weaker symptoms of dehydration than the wild-type after 30 days of no irrigation (Figure 7E). Moreover, the transgenic lines recovered more rapidly from drought than the wild-type after re-watering (Figure 7F). These results indicated that MeTPS1 was an active TPS and contributed to drought stress tolerance.

## 3. Discussion

### 3.1. Trehalose Contents in Different Plant Species

Trehalose is associated with stress resistance in many organisms [43,46]. However, trehalose level in angiosperm plants is extremely low, and has only been detected in significant amount in a few desiccation-tolerant plant species [22,47,48]. Cassava has been widely considered as a drought-tolerant crop [4,5,35], and a couple of mechanisms for drought tolerance have been proposed [3,6]. We detected high level of trehalose in cassava, *Jatropha*, and castor bean with concentrations of 0.75, 1.14, and 0.96 mg·g^−1^ FW in leaves, respectively, under normal conditions, whereas the other species that we tested (Figure 2) contained extremely low or undetectable levels of trehalose. Cassava, *Jatropha*, and castor bean all belong to the Euphorbiaceae family, and can be grown in arid, semi-arid and marginal lands, and thus are considered as drought-tolerant crops compared to other species [4,5,35]. Therefore, it is reasonable to assume that trehalose was responsible for drought tolerance in cassava, *Jatropha*, and castor bean.

The role of trehalose in drought stress tolerance in higher plants has been demonstrated in many species [26,27,28,49,50,51]. *Arabidopsis* contained a trehalose concentration of 0.01 mg·g^−1^ FW [26]. When trehalose synthesis was enhanced by introducing a trehalose-6-phosphate synthase gene (*35S::AtTPS1*) into the *Arabidopsis* genome, the trehalose concentration was increased to 0.026 mg·g^−1^ FW, and then dehydration tolerance was enhanced [26]. Transgenic tobacco expressing a yeast *TPS* gene accumulated a maximum of 0.015 mg·g^−1^ FW trehalose, and showed greater drought resistance compared to the wild-type [28]. Transgenic rice accumulating 0.017 mg·g^−1^ FW trehalose was also enhanced in abiotic stress tolerance, including drought [27]. Thus, trehalose was believed to be a stabilizer of biological structures and a positive regulator of stress tolerance [26,27]. The levels of trehalose that accumulated in non-transgenic cassava, *Jatropha*, and castor bean were much higher than the levels in transgenic *Arabidopsis*, tobacco, and rice, supporting the role of trehalose in natural drought tolerance in cassava, *Jatropha*, and castor bean.

However, the levels of trehalose in cassava, *Jatropha*, and castor bean were below the levels in some resurrection plants, even if trehalose level was increased up to approximately 2.6 mg·g^−1^ FW upon osmotic stress by PEG. In contrast, some resurrection plants, such as *Myrothamnus flabellifolius* or *Selaginella lepidophylla*, easily accumulated up to 10 mg·g^−1^ FW [29,52]. Therefore, the role of trehalose in cassava is potentially more complicated than that in resurrection plants.

### 3.2. Circadian Oscillation of Trehalose Level in Cassava Leaves

We observed circadian changes of trehalose level in cassava leaves (Figure 1C), and trehalose reached its highest level at noon (1:00 p.m.) when osmotic stress was a serious environmental problem that cassava leaves had to face with, implicating that trehalose played a role in drought stress tolerance in cassava and the circadian changes of cassava physiological processes. Circadian clocks are time-keeping mechanisms that coordinate the physiology of an organism to its surrounding environments. The interaction between the circadian clock of higher plants and their metabolic and physiological processes under environmental changes has been well studied [33,53]. One of the most important environmental changes is mid-day drought, which plants have to cope with [33,53]. Adjustments of the levels of primary and secondary metabolites appear to be firmly interconnected process in plants [33,53]. The most direct candidates for signal metabolites are sugars, including sucrose, glucose, fructose, and trehalose [10,54]. In the case of cassava, trehalose serves as an important candidate.

### 3.3. The Level of Trehalose, Rather than Fructose, Glucose and Sucrose, Is More Correlated with Dehydration Tolerance

We measured the soluble sugar contents in 41 cassava varieties using HPLC-ELSD. High levels of trehalose were recorded in all cassava varieties (Figure 1 and Figure 3). It is difficult to find a cassava variety as a trehalose negative control (with undetectable level of trehalose), and/or an apparent drought sensitive variety in cassava germplasm, since most cassava varieties tolerate a long period (2–3 months) of drought once the crop is established [4].

However, we managed to prove the importance of trehalose in dehydration tolerance by analyzing the correlation between soluble sugar contents and water retaining capacities in leaves of different varieties. Leaf morphology and surface area are diversified among cassava varieties. Our preliminary experiments indicated that leaf morphology and size had notable influence on water loss of detached leaves, which is consistent with previous research in other crops [55,56]. To focus on the role of soluble sugars, thirty-one cassava varieties with similar leaf morphology and surface areas were selected to study the relationships between soluble sugar concentrations and water retaining capacities of detached leaves (Figure 3). Trehalose had the highest correlation coefficient with water retaining capacity, compared to fructose, glucose, and sucrose (Figure 3). Fructose had significant but smaller correlation coefficient with water retaining capacity. Sucrose was the most abundant soluble sugar in all varieties (Figure 3); however, the correlation coefficient between sucrose and water retaining capacity was the lowest and was statistically not significant (Figure 3). These results suggested that trehalose is a more important osmolyte and/or signal transduction regulator than sucrose, glucose, and fructose in osmotic stress tolerance in cassava.

### 3.4. Osmotic-Stress Stimulates Trehalose Accumulation in Cassava

PEG 6000 has been widely used to simulate drought stress and stimulates a series of physiological changes [6,27]. We used 20% PEG in cassava, and observed physiological and transcriptional changes similar to drought stress [6]. In this research, we found a significant increase of trehalose concentrations in the leaves and the stems of PEG treated cassava plants at 1% level of significance (Figure 4A). In contrast, other soluble sugars including glucose, fructose, and sucrose did not show consistent changes within 24 h of osmotic stress at the same significant level (Figure 4B–D). This phenomenon is consistent with the results from rice, in which trehalose levels increased 2–3 folds in both wild-type and transgenic rice under drought-stress conditions, while glucose, fructose, and sucrose levels were almost unchanged [27]. These results further proved that trehalose is a more active player in osmotic stress response than other soluble sugars.

### 3.5. Gene Family of Cassava Trehalose Pathway Enzymes

The biosynthesis pathway of trehalose involves a two-step reaction in plants [44,52,57]. In the first step, trehalose-6-P is synthesized from UDP-glucose and glucose-6-P (G6P) catalyzed by trehalose-6-P synthase (TPS); in the second step, trehalose-6-P is dephosphorylated by trehalose-6-P phosphatase (TPP). Trehalose is then hydrolyzed to glucose by trehalase (TRE). Totally, we identified 12 putative *TPS* genes, ten putative *TPP* genes, and two *TRE* genes from the cassava genome databases.

TPS proteins in *Arabidopsis*, paddy rice, and maize were classified into two clades [37,38,39], and AtTPS1 and OsTPS1 have been demonstrated to be the only active TPS enzyme in *Arabidopsis* and paddy rice, respectively, while the remaining TPS proteins lack TPS activity [38,39]. Cassava TPS proteins were also classified into the two clades together with relevant TPS from maize, paddy rice, and *Arabidopsis* (Figure 5A). Interestingly, there were four cassava TPS proteins (MeTPS1–4) that were classified together with AtTPS1 and OsTPS1 in the same subclass (Figure 5A), which implies that MeTPS1–4 are potentially active TPS proteins, and cassava has potentially a larger number of active TPS than *Arabidopsis* and paddy rice. Moreover, we proved that *MeTPS1* was active by expressing it in tobacco (Figure 7). It is not known whether the class II cassava TPS proteins (MeTPS5–12) were active TPS. All Class II TPS proteins in *Arabidopsis* and rice have been shown to be inactive, although they all possess full TPS and TPP domains and the motifs required for TPS and TPP activities [38,39]. Therefore, MeTPS5–12 are most probably inactive in terms of TPS activity. However, they may be involved in regulatory function [38,39]. Interestingly, the active TPS proteins of AtTPS1 and OsTPS1 lacked the first motif required for the catalytic activity of TPP in the TPP domain [37,38,39]. It seems that the loss of TPP activity in plant TPS enzymes is essential for TPS activity. The four cassava Class I TPS: MeTPS1–4 that were classified in the clade of AtTPS1 and OsTPS1 lacked the first TPP motif similar to AtTPS1 and OsTPS1 (Figure S1), while MeTPS5–12 contained the motif (Figure S1). Therefore, cassava contained more active TPS proteins than *Arabidopsis* and rice.

Plants also contained a family of TPP proteins. Unlike the TPS family in plants, all the TPP genes in *Arabidopsis* encode active enzymes as revealed by heterologous expression in yeast (*Saccharomyces cerevisiae*) [58]. The TPP family in rice, maize, and *Arabidopsis* were classified into two clades [37,58]. However, cassava TPP proteins were classified into three clades (Figure 5B). Four (MeTPP1–4) were located in Clade A, and four (MeTPP5–8) in Clade B. Interestingly, MeTPP9 and MeTPP10 were not grouped with any TPP proteins from *Arabidopsis*, rice, and maize, and formed an independent clade, designated as Clade C. Moreover, the three motifs required for TPP activity in TPP proteins of cassava were more conserved than those in *Arabidopsis*, rice, and maize (Figure S2), especially the first motif: DXDX(T/V)(L/V/I) [43,44], where a single form DYDGTL was used. These results suggested that cassava may have enhanced TPP activity compared to *Arabidopsis*, rice, and maize.

Taken together, the gene families in cassava trehalose biosynthesis pathway are special, and the biosynthesis of trehalose in cassava may have been enhanced in the evolutionary history by growing in arid and marginal areas.

## 4. Materials and Methods

### 4.1. Plant Materials and Growth Conditions

Cassava varieties SC124, SC5, and 39 other randomly selected varieties were obtained from the Institute of Tropical Crops Genetic Resources, Chinese Academy of Tropical Agricultural Sciences (CATAS, located in Danzhou, Hainan, China), and planted in a field at the Institute of Tropical Bioscience and Biotechnology, CATAS located in Haikou, Hainan Province.

To study soluble sugars in different plant species, cassava (*Manihot esculenta*, variety SC124), *Jatropha curcas* variety 18, rubber tree (*Hevea brasiliensis*, variety 7-33-97), castor bean (*Ricinus communis*), tobacco (*Nicotiana benthamiana*), sweet potato (*Ipomoea batatas*), paddy rice (*Oryza sativa*) variety Teyou458, Cavendish banana (variety Williams), and sugarcane (*Saccharum officinarum*, variety ROC22) were grown in the field of Haikou campus, Chinese Academy of Tropical Agricultural Sciences (CATAS). Healthy mature leaves were randomly collected from six-month-old plants of cassava, *Jatropha*, rubber tree, banana, sugarcane, and castor bean, and/or from three-month-old plants of tobacco, potato, and paddy rice in the morning, when the weather was fine and the temperature was about 30 °C. The samples were collected in three replicates and stored at −80 °C until use.

To study soluble sugars in cassava varieties, stem stocks of 39 varieties were grown to about one meter in height (about 6 months), three leaves (the second, the third and the fourth full-expanded leaves from the apical meristem) were collected from each plant. Leaf samples of all 39 varieties were collected in the morning, when the weather was fine and the temperature was about 30 °C. The samples were collected in three replicates and wrapped in aluminum foil, then rapidly frozen in liquid nitrogen, and stored at −80 °C prior to analysis.

To study the distribution of trehalose in cassava plant, leaves, stems, and roots were collected from three-month-old plants of cassava variety SC5; To study the circadian oscillation of trehalose contents, leaves were collected from three-month-old plants of cassava varieties SC124 and SC5 at 8:00 a.m., 1:00 p.m., and 6:00 p.m. The samples were collected in three replicates and stored at −80 °C as described above.

### 4.2. Extraction and Purification of Soluble Sugars

Plant samples were ground in liquid nitrogen. Approximately 1 g powder was transferred into a 10 mL centrifuge tube, and 5 mL of hot distilled water was immediately added to the powder. The tubes were boiled at 100 °C for 15 min, and cooled to room temperature, and then centrifuged at 10,000× *g* for 10 min. The supernatant was transferred to a clean tube. The pellet was extracted twice with 2 mL hot distilled water. The supernatants were combined and dried at 80 °C. The residue was dissolved in 50% (*v*/*v*) acetonitrile to a final volume of 10 mL, and stored at 4 °C until use. The samples were filtered with 0.22 µm MillexTM membrane (Millipore Corporation, Bedford, MA, USA) three times before loading into the HPLC column.

### 4.3. High-Performance Liquid Chromatography with Evaporative Light-Scattering Detector (HPLC-ELSD) for Soluble Sugar Analysis

To prepare the standard curves for identification of glucose, fructose, and trehalose, 0.2 g standard substances (Sigma, St. Louis, MO, USA) were dissolved in 50% (*v*/*v*) acetonitrile to a volume of 50 mL. These solutions were diluted to prepare 0.1, 0.2, 0.5, 1.0, 2.0, and 4.0 mg·mL^−1^ standard solutions, and stored at 4 °C until use. To prepare the standard curve for identification of sucrose, 1, 2, 5, 10, 15, and 20 mg·mL^−1^ standard solutions were prepared. Ten microliters of each standard solution was loaded onto a Waters e2695 High Performance Liquid Chromatography instrument fitted with Empower software (Waters, Milford, CT, USA). The column was an XBridge-NH_2_ (Waters, Milford, CT, USA), and the column temperature was set to 25 °C. The mobile phase was acetonitrile: water (70:30, *v*/*v*) supplemented with 0.1% (*w*/*v*) NH_4_OH, and the flow rate was set to 1 mL·min^−1^. The eluate was detected and measured with an Alltech 3300 Evaporative Light Scattering Detector (ELSD; Alltech, Chicago, IL, USA). The ELSD drift-tube temperature was set to 85 °C, and the nitrogen flow velocity was 2 L·min^−1^. The regression equations and the correlation coefficients (*R^2^*) for the sugar quantity and the peak area were calculated using Excel 2007 (Chinese edition, Microsoft (China) Co., Ltd., Shanghai, China). To measure the concentrations of soluble sugars in the experimental samples, 10 µL of each sample was loaded onto the column and analyzed as described above. The concentrations of fructose, glucose, sucrose, and trehalose were calculated according to the regression equations.

### 4.4. Osmotic-Stress Treatment with Polyethylene Glycol (PEG 6000) Solution

Osmotic stress treatment of cassava was performed as previously described [6]. Stem segments of approximately 15 cm in length from cassava cultivar SC5 were planted in pots with a mixture of 50% vermiculite and 50% sand matrix. The plants were grown in greenhouse and watered once a day, and fertilized with 1/2 Hoagland solution [59] once a week. Forty-five days later, plants of similar growth stage (e.g., about 50 cm height and with 6–8 leaves) were selected and subjected to osmotic-stress treatments. Eighteen plants of similar size were selected and divided into three groups. Two groups of plants were treated with 20% PEG 6000 in soil for 12 and 24 h, respectively [6], and the third group was treated with water and was used as control. The leaves, stems, and roots were collected, and the soluble sugars were analyzed as described above. The significance of the differences between treatments was assayed by one-way ANOVA and LSD (Least Significant Difference) test using IBM SPSS Statistics Version 24.0 (IBM Corporation, New York, NY, USA).

### 4.5. Correlation of Trehalose Concentration and Dehydration Tolerance among Cassava Varieties

Dehydration tolerance of cassava varieties was evaluated by measuring water retaining capacity of detached leaves. Preliminary experiments showed that cassava leaf morphology and surface area is a significant factor that influenced water retaining capacity of detached leaves, therefore, 31 out of the 39 cassava varieties that had similar leaf morphology and size were selected to avoid unexpected influence on water loss. The field was irrigated one day before sampling. The third fully expanded leaf from the top of each plant was collected in the morning, and three leaves were collected for each variety. The leaves were weighed immediately (Wb), and placed separately on a long bench to allow equal atmospheric exposure. Room temperature was set to 25 °C, and humidity was 75%. The leaves were weighed at 30 min intervals (Wi) until withered. The leaves were finally dried to constant weight (Wt) in a constant-temperature dryer DKM812C (Yamato, Japan) at 80 °C. Water retaining capacity was calculated as relative water content in the detached leaves at 3 h after detachment, using the equation (Wi − Wt)/(Wb − Wt) × 100%. The experiments were replicated three times. The significance of the correlations between soluble sugar contents and water retaining capacities of detached leaves was analyzed using IBM SPSS Statistics Version 24.0, and the correlation curves in Figure 3 were constructed with Excel 2007.

### 4.6. Phylogenetic Analysis of the Trehalose Pathway Genes

The gene family members involved in trehalose biosynthesis and hydrolysis were mined in the Phytozome cassava genome database Version 4.1 accessed on 14 May 2015 (available at: ftp://ftp.jgi-psf.org/pub/compgen/phytozome/v9.0/Mesculenta/). A total of 25 putative trehalose pathway genes, including 12 putative trehalose-6-phosphate synthase (TPS) genes, ten putative trehalose-6-phosphate phosphatase (TPP) genes, and three trehalase (TRE) genes were identified from the cassava genome databases (Table S1). Reference sequences of *Arabidopsis*, rice, and maize were obtained from GenBank (available at: http://www.ncbi.nlm.hih.gov/) and Gramene (available at: http://ensemble.gramene.org) databases. The nomenclature for the genes in *Arabidopsis*, rice, and maize was adopted from previous publications [39,41,42,58], and gene symbols were provided in Table S1. The gene symbols for TPS and TPP genes in cassava were given sequentially according to phylogenetic analysis results and were listed in Table S1. For phylogenetic analysis, the amino acid sequences were aligned with ClustlX2 [41], and phylogenies were built using Maximum Likelihood (ML), Neighbor-Joining (NJ), and Minimum-Evolution (ME) methods integrated in MEGA 7 [42], and were tested using Bootstrap method (1000 replicates). The phylogenies constructed with ML, NJ, and ME methods were similar, and only the ML trees are shown (Figure 5). The confidence probabilities (multiplied by 100) that the interior branch length is greater than 0 are presented at the relevant nodes. Scale bars represent 0.1 residue substitution.

### 4.7. 5′ RACE (Rapid Amplification of cDNA Ends) of MeTRE2

To determine 5′ end of a truncated trehalase gene *MeTRE2* (cassava4.1_015916m.g), total RNA was extracted from leaves of cassava variety SC8 using a Plant RNA Extraction Kit (TaKaRa Biotechnologies, Dalian, China). mRNA was purified using a Dynabeads mRNA purification kit (Invitrogen, Beijing, China). First-strand cDNA was synthesized using a 5′ Full RACE kit with TAP (TaKaRa) accordingly to the manufacturer’s protocol. Two rounds of RACE PCR were performed using a gene specific primer and a universal primer each provided in the kit. For the first round PCR, the *MeTRE2* gene specific primer was 5′-CAATTCGGGTTGCTTCCTGACTTCG-3′ that was designed with MacVector 14.0 (MacVector Inc., Apex, NC, USA). For the second round PCR, the *MeTRE2* gene specific primer was 5′-CTTAGGCAAGAACCCATCAGGCTC-3′. The PCR reactions were carried out using the parameters suggested by the manufacturer.

### 4.8. Differential Expression Analysis of Trehalose Pathway Genes

The influence of PEG-simulated drought stress on cassava was studied previously, and a large transcriptome dataset of leaves (folded leaf, functional leaf, and bottom leaf) and roots before and after 20% PEG treatment was generated using RNA-seq method [6], and a novel pathway in ABA-dependent and ABA-independent regulatory networks underlying PEG-induced dehydration response were detected [6]. However, trehalose pathway genes were not investigated [6]. This dataset was used to investigate the differential expression of trehalose pathway genes upon PEG stress in this research. The expression levels of the trehalose pathway genes in all samples in TPM (Transcripts Per kilobase Million) were compiled with Excel 2007, and were normalized using logarithmic method with two as base. The normalized dataset was then exported to HemI toolkit [45]. Heatmap was built using the default parameters.

### 4.9. Transformation of MeTPS1 into Tobacco

*MeTPS1* coding sequence was amplified with primers 5′-CGGGATCCATGCCTGGAAACCAGTATAACG-3′ and 5′-GCGTCGACTCAAGAAGATGCCCTGGCTAG-3′, and cloned in the pEASY-T1 Simple cloning vector (TransGen Biotech, Beijing, China). The plasmid with correct sequence was digested with *Bam* HI and *Sal* I, and ligated into pCAMBIA2300-35S-OCS [60], resulting in the expression vector pCAMBIA2300-*MeTPS1*. After confirmation by sequencing, the vector was transformed into *Agrobacterium tumerfaciens* GV3103 via electroporation. Seeds of *Nicotiana benthamiana* were sterilized for 30 s in 70% (*v*/*v*) ethanol, and then 5 min in 5% bleach, followed by rinsing with sterile distilled water for three times. The seeds were then sown on half strength semi-solid MS medium supplemented with 30 g·L^−1^ sucrose and 6 g·L^−1^ agar (Beijing Solarbio Science & Technology Co., Ltd., Beijing, China). Plants were incubated at 16 h light per day with light intensity of 3000 Lux. Twenty-day-old seedlings were used for transformation using leaf discs as explants according to previously described [61]. The infected explants were cultured on the selection medium supplemented with 2.0 mg·L^−1^ 6-benzylaminopurine (6-BA), 0.5 mg·L^−1^ indole-3-acetic acid (IAA), 10 mg·L^−1^ hygromycin, and 500 mg·L^−1^ carbenicillin. The explants were transferred to fresh selection medium once every two weeks. Shoots resistant to hygromycin were transferred to the rooting medium (MS supplemented with 15 mg·L^−1^ hygromycin and 250 mg·L^−1^ carbenicillin). Plants with high quality roots were used to test the presence of foreign gene by PCR.

The putative transgenic plants were transplanted in a mixed soil (50% soil and 50% coconut husk) and incubated in the green house until they flowered and set seeds (T1). The T1 seeds were then sterilized and grown on selection medium again to screen for plants that inherited the foreign gene. The selected plants were grown in green house until seeds of T2 generation were set. The T2 seeds were sterilized and sown in selection medium, and the lines with 100% transgenic seeds were considered homozygous and were kept for further analysis.

### 4.10. Confirmation of the Transgenic Tobacco Plants by PCR and RT-PCR

Genomic DNA was isolated from transgenic and wild type tobacco plants using a Universal Genomic DNA Extraction Kit (TaKaRa Biotechnologies, Dalian, China). Total RNA was extracted according to a Plant RNA Extraction Kit (TaKaRa Biotechnologies, Dalian, China). The quality of total RNA was evaluated with a 2100 Bioanalyzer (Agilent Technologies, Santa Clara, CA, USA). The mRNA was reverse transcribed to cDNA using a Superscript III First-Strand Synthesis System (Invitrogen, Carlsbad, CA, USA). PCR was performed using *MeTPS1* gene-specific primers 5′-ATGCCTGGAAACCAGTATAACG-3′ and 5′-TCAAGAAGATGCCCTGGCTAG-3′. Amplifications were performed using a T-1 Thermocycler (Biometra GmbH, Göttingen, Germany) at 94 °C for 4 min, followed by 35 cycles of 94 °C for 30 s, 55 °C for 30 s, and 72 °C for 90 s, and a final extension at 72 °C for 5 min. The PCR products were visualized with a Tanon 4100 Gel Imaging System (Tanon Science & Technology Co., Ltd., Shanghai, China) after electrophoresis.

## Supplementary Materials

Supplementary materials can be found at http://www.mdpi.com/1422-0067/17/7/1077/s1.
