# Supplementary material for: Interspecies and Intraspecies Analysis of Trehalose Contents and the Biosynthesis Pathway Gene Family Reveals Crucial Roles of Trehalose in Osmotic-Stress Tolerance in Cassava
Source: Int J Mol Sci. 2016 Jul 13;17(7):1077. doi: 10.3390/ijms17071077 (PMC4964453; doi:10.3390/ijms17071077)
Supplement: Supplementary file 1 [file ijms-17-01077-s001.pdf]

Supplementary Materials: Interspecies and Intraspecies Analysis of Trehalose Contents and the Biosynthesis Pathway Gene Family Reveals Crucial Roles of Trehalose in Osmotic-Stress Tolerance in Cassava

Bingying Han, Lili Fu, Dan Zhang, Xiuquan He, Qiang Chen, Ming Peng and Jiaming Zhang

```
MeTPS1     1 MISKQLLTERYAPCQGLDVMSCKLQASSQNKNNCTLVTHKTGLESLLLDRVRKFTELFAN  60
MeTPS2     1 MPGNKYNGTPVTPRTRLERLLLNRELRKFNR------FFNPSDE---LDDGLKEAELFAN  51
MeTPS3     1 MPGNQYNGNSTPYPSRTQRLLRERELKKSSRASHSNEVTDNHGGTEPCEHDLRLRERDNS  60
MeTPS4     1 MPGNQYNSNSTPYPSRTQRLLRERELKKSSRSSHSNEATDNHRGIEPCEHDLRLREGDDS  60
MeTPS5     1 MVSRSCMNFLDLASGNLLEIP---------------------------HTPRSIPRVMTV  33
MeTPS6     1 MVSRSCLNILDLASGNLLEIP---------------------------HTPRSIPRVMTV  33
MeTPS7     1 MMSRSCINLLDLASGDMLNLP---------------------------RTPRSLPRVMTV  33
MeTPS8     1 MMSKSCINFLDLSSGDMLNFS---------------------------RTPRSLPRVMTV  33
MeTPS9     1 MMSRSYTNLLDLASGNFPVMGQP-------------------------REKKRLPRVMTV  35
MeTPS10    1 MVSRSYSNLLDLASGESPTFG---------------------------RERKRLPRVATV  33
MeTPS11    1 MVSRSYSNLLELASGESPSFG---------------------------RMTRRIPRIMTV  33
MeTPS12    1 MVSRSYSNLLELASGESPSFG---------------------------RMSRRIPRIMTV  33

                                                 GG1/GP1
MeTPS1    61 DSVTKETETHGPLHEDTSLEEVNVCERPRRPKQRLLVVANSLPVSAIRKGQ--DSWKLEI 118
MeTPS2    52 DSVLNKDENCGPFNEETFSEGVDGSER--LHKQRLLVVANRLPVSAVRKGE--DSWQLEM 107
MeTPS3    61 NNSYIDQYVEGAIAATRQLAEGCEKQDGRPLRQRLLVVANRLPVSAVRRGE--DSWSLEI 118
MeTPS4    61 NNCYIEQDLEGAIAATKTLAEGCEYQDARPLRQRLLVVANRLPVSAVRRGE--DSWSLEI 118
MeTPS5    34 PGIISDLDGYGPY---GSNDEDSEIA-SSICRERKIIVANMLPLQAKKDPG-TAKWCFSW  88
MeTPS6    34 PGIISDLDGYG------SNDGDSENA-SSICRERIIIVANMLPLHAKKDPE-TAKWFFSW  85
MeTPS7    34 PGIFSDTDGDG------SNDGDSDAP-SSGCGKKQIIVANFLPLHAQKDLN-SSKWSFSF  85
MeTPS8    34 EGIFSNTDSDG------SNDGDTNVP-SARCYKKKIIVANFLPLHAQKDLE-TSKWSFSF  85
MeTPS9    36 PGVISELDDDQ------ANSVASDVP-SSLVQDRIIIVANQLPVKAKRRPD-NKGWGFSW  87
MeTPS10   34 AGVLSELEDEN------SNSVGSDAP-SSVSQERMIIVGNQLPLRVHQSPDGNGEWCFSW  86
MeTPS11   34 AGIISDLDDDP------SASVCSDPSSSSIQKDRIIIVANQLPIRAQRKSDGSKSWIFTW  87
MeTPS12   34 AGIISDLDDDP------TDSVCSDPSSSSVQRDRIIIVANQLPIRAQRKSDGSKSWIFTW  87

               UR1
MeTPS1   119 SVDGLVNPL---LGVKEFEARWIGWAGVNVPDEIGQKALSTALAEKKCIPVFLDEHIVHQ 175
MeTPS2   108 SVGGLVSAL---LGVKEFDARWIGWAGVNVPDEIGQKALTTALAEKRCIPVFLDEDIVHQ 164
MeTPS3   119 SAGGLVSAL---LGVKEFEARWIGWAGVNVPDEIGQKALTKALAEKRCIPVFLDEEIVHQ 175
MeTPS4   119 SAGGLVTAL---LGVKEFEARWIGWAGVNVPDEIGQKALTRALAEKRCIPVFLDEEIVHQ 175
MeTPS5    89 DEDSLFLQLKDGFS-PETEVIYVGSLKADIDATDQEEVSQKLLEDFNCVPTFLPQDLQRK 147
MeTPS6    86 DEDSLYLQLKDGLS-PETEVIYVGSLKADIKASEQEEVSQQLLENFNCVPTFLPQDLQKK 144
MeTPS7    86 DEDSILLQMKDGFS-INTEVVYVGSLKVDVDTSEQEEVSQKLLEEFNCVPTFLPPDLHKK 144
MeTPS8    86 DEDSLLFQMKDGFS-DNTEIVYVGSLKVDVDASEQEEVSQKLLEEYNCVPTFLPPELHKK 144
MeTPS9    88 DEDSLLLQLKDGLP-EDMEVIYVGSLRVEVDLSEQDDVSQLLLDRFKCVPAFLPPDILSK 146
MeTPS10   87 DEDSLLLQLRDGLG-EDVEVIYVGCLKEEVDPSEQDDVAQTLLESFKCVPAFIPPELFSK 145
MeTPS11   88 DENSLLLQLKDGLGDDDIEVIYVGCLREDIHPSEQDEVSQILLETFKCVPTFLPPDLFSR 147
MeTPS12   88 DENSLLLQLKDGLGDDDIEVIYVGCLREEIHPSEQDEVSQILLETFKCVPTFLPPDLFSR 147
```

Figure S1. *Cont.*

```
             GP2      GG2
MeTPS1   176 YYNGYCNNVLWPLFHYLGLPQEDRLPTTRSFQSQFDAYKKANQMFADVVNRHYE-EGDVV 234
MeTPS2   165 YYNGYCNNMLWPLFHYLGLPQEDRLATTRSFQSQFDAYKKANQMFADVVNKHYK-EGDVV 223
MeTPS3   176 YYNGYCNNMLWPLFHYLGLPQEDRLATTRSFQSQFAAYKKANQMFADVVNKHYE-EGDVV 234
MeTPS4   176 YYNGYCNNILWPLFHYLGLPQEDRLATTRSFQSQFAAYKKANQMFADVVNKHYE-EGDVV 234
MeTPS5   148 FYLGFCKQQLWPLFHYMLPMCPD--HGDRFDRNLWQAYVSANKIFADKVMEIISPDEDYV 205
MeTPS6   145 FYLGFCKQQLWPLFHYMLPMCPD--HGDRFERVVWQAYVSANKMFADKVMEIISPEEDYV 202
MeTPS7   145 FYHGFCKHQLWPLFHYMLPMFPD--HGERFDKLLWQAYVSANKVFADKVMEVINPEEDYV 202
MeTPS8   145 FYHGFCKHQLWPLFHYMLPMCPD--HSNRFDKLLWQAYVSANKIFADKVMEVINPEEDHV 202
MeTPS9   147 FYHGFCKQHLWPLFHYMLPFSAS--HGGRFDRSLWEAYVAANKIFSQRVVEVLNPEDDYV 204
MeTPS10  146 YYHGFCKQHLWPLFHYMLPLSPD--LGGRFDRSLWQAYVSVNKIFADKVMEVISPDDDYV 203
MeTPS11  148 YYHGFCKQQLWPLFHYMLPLSPE--LGGRFNRSLWQAYVSANKIFADRIMEVINPEDDFV 205
MeTPS12  148 FYHGFCKQQLWPLFHYMLPLSPD--LGGRFNRSLWQAYVSANKIFADRIMEVINPEDDFV 205

               GG3 GP3                  UG1 GG4                        UG2
MeTPS1   235 WCHDYHLMFLPKCLKEYNSKMKVGWFLHTPFPSSEIHRMLPSRSELLRSVLAADLIGFHT 294
MeTPS2   224 WCHDYHLMFLPKCLKEYNSKMKVGWFLHTPFPSSEIHRMLPSRSELLRSVLAADLVGFHT 283
MeTPS3   235 WCHDYHLMFLPKCLKEYNSNMKVGWFLHTPFPSSEIHRTLPSRSELLRSVLAADLVGFHT 294
MeTPS4   235 WCHDYHLMFLPKCLKEYNSNMKVGWFLHTPFPSSEIHRTLPSRSELLRSVLAADLVGFHT 294
MeTPS5   206 WIHDYHLMLLPTFLRKGYNRVKLGFFLHSPFPSSEIYRTLPVRDEILRGLLNCDLIGFQT 265
MeTPS6   203 WVHDYHLMLLPTFLRKAYNRVKLGFFLHSPFPSSEIYRTLPVRDEILRGLLNCDLIGFHT 262
MeTPS7   203 WVHDYHLMVLPTFLRKRFYRIKLGFFLHSPFPSSEIYRNLPVRDEILKALLNADLIGFHT 262
MeTPS8   203 WVHDYHLMVLPTFLRKHFYRVKLGFFLHSPFPSSEIYRTLPVRDEILKALLNADLIGFHT 262
MeTPS9   205 WIHDYHLMVLPTFLRRRFNRLRMGFFLHSPFPSSEIYRTLPVREEILKALLNSDLIGFHT 264
MeTPS10  204 WVHDYHLMVLPTFLRKRFNRVKLGFFLHSPFPSSEIYRTLPVRDELLRALLNADLIGFHT 263
MeTPS11  206 WVHDYHLMVLPTFLRKRFNRVKLGFFLHSPFPSSEIYKTLPIREELLRALLNSDLIGFHT 265
MeTPS12  206 WVHDYHLMVLPTFLRKRFNRVKLGFFLHSPFPSSEIYKTLPIREELLRALLNSDLIGFHT 265

MeTPS1   295 YDYARHFVSACTRILGLEGTP----EGVEDQGKLTRVAAFPIGIDSDRFIRALELPQVQD 350
MeTPS2   284 YDYARHFVSACTRILGLEGTP----EGVEDQGKLTRVAAFPIGIDSERFIRALELPQVQD 339
MeTPS3   295 YDYARHFVSACTRILGLEGTP----EGVEDQGRLTRVAAFPIGIDSDRFIRALEAPQVQE 350
MeTPS4   295 YDYARHFVSACTRILGLEGTP----EGVEDQGRLTRVAAFPIGIDSDRFIRALEVPQVQD 350
MeTPS5   266 FDYARHFLSCCSRMLGLDYESKRGHIGLDYFGRTVYIKILPVGIHVCRLESVMNLPYTSA 325
MeTPS6   263 FDYARHFLSCCSRMLGLDYESKRGHIGLDYFGRTVYIKILPVGIHMGRLESVMNLPSTSV 322
MeTPS7   263 FDYARHFLSCCSRMLGLDYESKRGHIGLEYFGRTVYIKILPVGVHMGRLESALNHPSSSI 322
MeTPS8   263 FDYARHFLSCCSRMLGLDYESKRGHIGLEYFGRIVYIKILPAGIHLGRLESALNHPSSSI 322
MeTPS9   265 FDYARHFLSCCSRMLGLEYQSKRGYIGLEYYGRTVGIKIMPVGIHMGQIESVLRLADKEW 324
MeTPS10  264 FDYARHFLSCCSRMLGLSYQSKRGYIGLEYYGRTVSIKILPVGIHIGQLQSVLNLPETES 323
MeTPS11  266 FDYARHFLSCCSRMLGLTYESRRGYIGLDYCGRTVSIKILPVGIHMGQLQSVLRLPETET 325
MeTPS12  266 FDYARHFLSCCSRMLGLTYESKRGYIGLEYCGRTVSIKILPVGIHMGQLQSVLRLPETET 325

                                 UP1   UP2                            GP4
MeTPS1   351 HIKELKDKFS--GRKVMLGVDRLDMIKGIPRKILAFEEFLEENPEWRDKVVLLQIAVPSR 408
MeTPS2   340 HIKELKERFA--GRKVMLGVDRLDMIKGIPQKILAFEEFLEENPEWRDKVVLLQIAVPTR 397
MeTPS3   351 HIKDLKERFA--GRKVMLGVDRLDMIKGIPQKILAFEKFLEENSHWRDKVVLLQIAVPTR 408
MeTPS4   351 HIKELKERFS--GRKVMLGVDRLDMIKGIPQKILAFEKFLEENSHWRDKVVLLQIAVPTR 408
MeTPS5   326 KVKEIQEQFS--GKKVILGIDDMDIFKGISLKLLAMEQLLQQHPDLQGKVVLVQIVNPAR 383
MeTPS6   323 KVKEIQEQLG--GRKVILGIDDMDIFKGISLKLLAMEQLLQQHPELRGKVVLVQIVNPAR 380
```

Figure S1. *Cont.*

```
MeTPS7    323 KVKEIKKQFE--GKRIIVGADDMDIFKGISLKLLAMEHLLQQNPELRGKVVMVQIVNPAR 380
MeTPS8    323 KVKEIQTQFE--GKKIIVGVDDMDIFKGISLKLLAMEQLLQQNPELRGKVVMVQILNPAR 380
MeTPS9    325 RVGELKQQFE--GKTVLLGVDDMDIFKGVNLKLLAMEQMLKQHPKWQGRAVLVQIANPAR 382
MeTPS10   324 KVAELHDQFR--GQTVILGVDDMDIFKGISLKLLAMEQLLLQHPDKRGEVVLVQIANPAR 381
MeTPS11   326 KVMKLIKQFGDRGRIMLLGVDDMDIFKGISLKLLAMEQLLVQHPEWRGKVVLVQIANPAR 385
MeTPS12   326 KVVELIKQFNDQGRIMLLGVDDMDIFKGISLKLLAMEQLLVQHPEWRGKVVLVQIANPAR 385

                                                     UU
MeTPS1    409 THVPEHQKLTSQVHKIVTRINGRFGTLTAVPIHHLDRTLDFHALCALYAVTDVALVTSLR 468
MeTPS2    398 TDVPEYQKLTSQVHEIVGRINGRFGTLTAVPIHHLDRSLDFHALCALYAVTDVALVTSLR 457
MeTPS3    409 TDVPEYQKLTSQVHEIVGRINGRFGTLTAVPIHHLDRSLDFHALCALYAVTDVALVTSLR 468
MeTPS4    409 TDVPEYQKLTSQVHEIVGRINGRFGTLTAVPIHHLDRSLDFHALCALYAVTDVALVTSLR 468
MeTPS5    384 GSGKDVQEAKRETYLIAKRINDVYGSHEYEPVILIDRPVPRYEKTAYYAVAECCIVNAVR 443
MeTPS6    381 GSGKDVQEAKRETYLTAKRINEVYGSPKYEPVILIDRPVPRYEKTAYYALAECCIVSAVR 440
MeTPS7    381 SSGKDVQEAKKETYSITKRINRIFGFPGYEPVVLIDRHVPFYEKTAYYALAECCIVNAVR 440
MeTPS8    381 SSGKDVQEAERETYLTTKRINSIFGFPGYDPVILIDRHVPLYEKTAYYSLAECCIVNAVR 440
MeTPS9    383 GKGKDLEEIQAEIQASCKRINETFGQPGYEPVVFIDRPVSLSERAAYYTVAECVVVAAVR 442
MeTPS10   382 GRGRDVQEVQSETKATVRRINEIFGRPGYAPVVLIDTPLQFYERIAYYVIAECCLVTAVR 441
MeTPS11   386 GRGKDVKEVQAETYSTVKRINEMFGKPGYDPVVLIDAPLKFYEKVAYYVVAECCLVTAVR 445
MeTPS12   386 GKGKDVKEVQAETYSTVKRINETFGKPGYDPIILIDAPLKFYEKVAYYVVAECCLVTAVR 445

                UG4-5
              UG3 UP3-5  UR2
MeTPS1    469 DGMNLVSYEFVACQ---------------TSKKGVLILSEFAGAAQSLGAGAILVNPWNI 513
MeTPS2    458 DGMNLVSYEFVACQ---------------ASKKGVLILSEFAGAAQSLGAGAILVNPWNI 502
MeTPS3    469 DGMNLVSYEFVACQ---------------DAKKGVLILSEFAGAAQSLGAGAILVNPWNI 513
MeTPS4    469 DGMNLVSYEFVACQ---------------DSKKGVLILSEFAGAAQSLGAGAILVNPWNI 513
MeTPS5    444 DGMNLVPYKYIVCRQGTPYMDKAMGITSDSPRTSMLVVSEFVGCSPSLSG-AIRVNPWDI 502
MeTPS6    441 DGMNLVPYKYIVCRQGTLYMDKAMGTASDSPRKSMIVVSEFIGCSPSLSG-AIRVNPWDI 499
MeTPS7    441 DGMNLIPYKYIVCRQGTPKMDEALGINHQLPHSSTIVVSEFIGCSPSLSG-AIRVNPWDV 499
MeTPS8    441 DGMNLIPYEYIVCRQGTPKMDEALGVDPRSSHASTIVVSEFIGCSPSLSG-AIRVNPWDV 499
MeTPS9    443 DGMNLTPYEYIVCRQGVSGSESSS--ESSGPKKSMLVVSEFIGCSPSLSG-AIRVNPWNI 499
MeTPS10   442 DGMNLIPYEYVICRQGNDKLDETLGLNTFDPKKSMLVVSEFIGCSPSLSG-AIRVNPWNI 500
MeTPS11   446 DGMNLIPYEYIISRQGNEQLDKVLRLEACTPKKSMLVISEFIGCSPSLSG-AIRVNPWNI 504
MeTPS12   446 DGMNLIPYEYIISRQGNERLDKVLRLEPSALKKSMLVISEFIGCSPSLSG-AIRVNPWNI 504

MeTPS1    514 TEVASSISYALNMPADERENRHNHNFRHVTTHTSQEWAATFVSELNDTIVEAQLR----- 568
MeTPS2    503 SEVASSIGYALNMPADEREKRHNHNFRHVTTHTSQEWAATFVSELNDTIVEAQLR----- 557
MeTPS3    514 TEVADSIRQALTMSCEEREKRHHHNFAHVTTHTAQEWAETFVSELNDTVIEAQLR----- 568
MeTPS4    514 TEVADSIRQALTMSCEEREKRHRHNFAHVKTHTAQEWAETFVSELNDTVIEAQLR----- 568
MeTPS5    503 DAVADALNLAITMRESEKQLRHEKHYRYVSTHDVAYWARSFMQDLERACQDHYNKRCWGI 562
MeTPS6    500 DAVADALNLAITMPESEKQLRHEKHYRYVSTHDVAYWARSFMQDLERACQDHYNKRCWGI 559
MeTPS7    500 EAVADALNLALTMPDLEKQLRHEKHYRYISSHDVAYWARSFMQDLERACKDHYSKRCWGI 559
MeTPS8    500 EAVADALNLALTMPDLEKQLRHEKHYRYISSHDVAYWVRSFVLDLERACKDHYSKRCWGI 559
MeTPS9    500 EATAEAMNEAISMSESEKQLRHEKHYRYVSTHDVAYWSKSFFQDMERTCKDHFRRRCWGI 559
MeTPS10   501 DAVAEAMDSALVIPEPEKQMRHEKHYRYVSTHDVAYWARSFLQDLERACRDHVRRRCWGI 560
MeTPS11   505 DAVADAMDYALEMAEPEKQLRHDKHYKYVSTHDVGYWARSFLQDLERTCRDHARRRCWGI 564
MeTPS12   505 DAVADAMDCALEMAEPEKQLRHDKHYKYVSTHDVGYWARSFLQDLERTCRDHARRRCWGI 564
```

Figure S1. *Cont.*

```
                                                  Motif I
MeTPS1    569 ----------TRQVLPLLPIHDAVERYLQSNNRLLILGFNATLTEPVDSLGRRSGQIREM 618
MeTPS2    558 ----------TRQVLPLLPVNDAVERYTQSSNRLLILGFNATLTEPVHTLGRRGSQIREM 607
MeTPS3    569 ----------TRQVPPILPEEDAIKRYLQSNNRLLILGFNATLTEPVDTPGRRVDQIKEM 618
MeTPS4    569 ----------TRQVPPILPEEDAIERYLQSNNRLLILGFNATLTEPVDTPGRRGDQIKEM 618
MeTPS5    563 GFGLGFRVVSLSPSFRKLCIEHIVSAYKRTNRRAIFLDYDGTVVPQTSIVKSPS------ 616
MeTPS6    560 GFGLGFRVVSLSPSFRRLGVEHIVSAYKRTNRRAIFLDYDGTVVPHTSIVKSPS------ 613
MeTPS7    560 GFGLNFRILSLSPSFRKLSNEHIVSAYKKTYRRVIFLDYDGTVVPQTSIVKTPS------ 613
MeTPS8    560 GFGLNFRILSLSPNFRKLSSEHIVSAYKRTYRRVIFLDYDGTVVPQTSIVKTPS------ 613
MeTPS9    560 GFSFGFRVVALDPNFRKLSTDAIVSAYLRSKNRAILLDYDGTVMPQTSINKTPS------ 613
MeTPS10   561 GFGLGFRVIALDPNFRKLSVEHIVSAYKRTKHRAILLDYDGTMILSGSISTAPS------ 614
MeTPS11   565 GFGLSFRVVALDPNFRKLSMEHIVSAYKRTTTRAILLDYDGTLMPQASIDKSPS------ 618
MeTPS12   565 GFGLSFRVVALDPNFRKLSMEHIVSAYKRTTTRAILLDYDGTLMPQASIDKSPS------ 618

                                       Motif II
MeTPS1    619 EPRLHPGLKEPLKKLCDDQMTTIVVLSGSDRTILDENFGGYN-MWLAAENGMSLRLTTG- 676
MeTPS2    608 EPRLHPQLKEPLKKLCKDQMTTVVVLSGSDRTILDENFGEYN-MWLAAENGMFLRRTTG- 665
MeTPS3    619 ELKLHPELKETLVALCSDPKTTIVVLSGSDSNVLDKNFGEFD-MWLAAEHGMFLRFTKG- 676
MeTPS4    619 ELKLHPELKETLVALCSDPKTTIVVLSGSERNVLDENFGEFD-MWLAAEHGMFLRLTKA- 676
MeTPS5    617 -----AEVISVLKTLCNDQNNTVFIVSGRGKNSLSEWLDPCERLGIAAEHGYFMRYNKTS 671
MeTPS6    614 -----PEVMSVLTTLCNDPNNTVFIVSGRARNSLSEWLDPCERLGIAAEHGYFMRWDKTC 668
MeTPS7    614 -----SEVISVLNNLCSDPKNTVFIVSGRGKDPLSDWFAQCENLGIAAEHGYLMRWNKTS 668
MeTPS8    614 -----PEVISVLSNLCTDPKNTVFIVSGRAKDTLSDWFTQCENLGIAAEHGYFMRWSRMS 668
MeTPS9    614 -----QEVISIINALCGDVKNTVFIVSGRGRESLGKWFSPCKKLGIAAEHGYFIRWSVDE 668
MeTPS10   615 -----TEAVGILNNLCRDPKNVVFVVSGKDKETLAEWFSSSEKLGIAAEHGYFVRPNHDV 669
MeTPS11   619 -----PKSIDILNSLCRDKNNMVFLVSARSRKTLTEWFSQCEKLGLAAEHGYFRRLTRDA 673
MeTPS12   619 -----SKSIDILNSLCRDKNNMVFLVSARSRKTLTEWFSQCEKLGLAAEHGYFLRLTRDA 673

MeTPS1    677 EWMTTMPENLNMDWVDSVKHVFEYFTERTPRAYYELRETSLVWNYKYADVEFGRLQARDM 736
MeTPS2    666 KWMTTMPENLNMDWVDSVKHVFEYFTERTPRSHFELRETSLLWNYKYADVEFGRLQARDM 725
MeTPS3    677 EWMTTMPEHLNMEWVDSVKHVFEYFTERTPRSHFELRETSLVWNYKYADVEFGRLQARDM 736
MeTPS4    677 EWMTTMPDHLNMEWVDSVKHVFEYFTERTPRSHFELRETSLVWNYKYADVEFGRLQARDM 736
MeTPS5    672 DWETNPVTDNDLDWKNIVEPIMRSYTEATDGSSIEVKESALVWHHQDADPDFGSCQAKEL 731
MeTPS6    669 EWETKSVAD-DLDWKNIVEPIMGLYTETTDGSNIELKESALVWHHQDADPDFGSCQAKEL 727
MeTPS7    669 SWETSSLVA-DFDWKRIAVPVIKLYTEATDGSYIETKESALVWHHQYADPDFGSCQAKEL 727
MeTPS8    669 NWETSNLVA-DFDWKKIALPVMKSYTEATDGSYIETKESALVWNHQYADPVFGSCQAKEL 727
MeTPS9    669 QWETCGQNS-DFGWIHIAEPVMKLYTESTDGSSIETKESALVWHHRDADPGFGASQAKEM 727
MeTPS10   670 DWETCVSVP-DFDWKQIAEPVMKLYTETTDGSAIETKESALVWNYQYADPDFGSCQAKEL 728
MeTPS11   674 EWETCVPVT-DTAWKQIAEPVMHLYTETTDGSTIEDKETAVVWCYEDADPDFGSCQAKEL 732
MeTPS12   674 EWETCVPVT-DIAWKQIAEPVMQLYTETTDGSTIEDKETALVWCYEDADPDFGSCQAKEL 732

                                               Motif III
MeTPS1    737 LQHLWTGPISNAAVDVVQGGRSVEVRAVGITKGAAIDRILRQIVHNQGMRTPIDYVLCVG 796
MeTPS2    726 LQHLWTGPISNAAVDVVQGRRSVEVRAVGVTKGAAIDRILGEIVHNQGMKAPIDYVLCVG 785
MeTPS3    737 LQHLWTGPISNASVDVVQGGRSVEVRAVGVTKGAAIDRILGEIVHSKSMTTPIDYVLCIG 796
MeTPS4    737 LQHLWTGPISNASVDVIQGSRSVEVRPVGVTKGAAIDRILGEIVHSKSMTTPIDFVLCVG 796
```

Figure S1. *Cont.*

```
MeTPS5    732 LDHLEN-VLANEPAVVKRGQHIVEVKPQGISKGSVAEKVLLDMVNHG---KPPDFVLCIG 787
MeTPS6    728 LDHLEN-VLANEPAVVKRGQHIVEVKPQGISKGFVAEKVLLNMVNRG---KPPDFVLCIG 783
MeTPS7    728 LDHLEN-VLANEPVVVKRGQHIVEVKPQGVTKGLVAERVLSAMINNG---KSPDFVMCIG 783
MeTPS8    728 LDHLET-VLANEPVVVKRGQHIVEVKPQGVTKGLVAEKVLYAMVTSG---KSPDFVMCIG 783
MeTPS9    728 LDHLES-VLANEPVAVKSGQFIVEVKPQGISKGFVAERIFTSMVERG---KQADFVLCIG 783
MeTPS10   729 LDHLES-VLTNEPVSVKSGQHIVEVKPQGVNKGLVAQCLLETMQKKG---MLPDFVLCIG 784
MeTPS11   733 LDHLES-VLANEPVTVKSGQNTVEVKPQGVSKGLVAKRLLSTMQEKG---MSPDFVLCIG 788
MeTPS12   733 LDHLES-VLANEPVTVKSGQNIVEVKPQGVSKGLVAKRLLSTMQERG---MSPDFVLCIG 788

MeTPS1    797 HFLAKDEDIYTFFEPEHPSESPGIVRTKS------------VPKVPYGGSRAQYHRQKQ- 843
MeTPS2    786 HFLVKDEDIYTFFEPELPIETPPIVRCRLPE-----PVGLPVPKIPCGRSRSKTHLKKQ- 839
MeTPS3    797 HFLGKDEDVYTFFEPVLPSDGIGIARAKQTDGLKSPGERRPSLKLPASKSGSKSSQGKTQ 856
MeTPS4    797 HFLGKDEDVYTFFEPVLPSDGVGIARTKQTDGLKSPGEKRPPPKLPA-KSGSKSSQGKS- 854
MeTPS5    788 DDK-SDEDMFGSILSTVSDPTLPVA----------------------------------- 811
MeTPS6    784 DDK-SDEDMFESILSTVSGPTLTVA----------------------------------- 807
MeTPS7    784 DDR-SDEDMFESISRTASSLSFCSA----------------------------------- 807
MeTPS8    784 DDR-SDEDMFESISRKGSSWSFNSA----------------------------------- 807
MeTPS9    784 DDR-SDEDMFEIIGNATANGVLSSS----------------------------------- 807
MeTPS10   785 DDR-SDEDMFEVIMSARAGPSLSPV----------------------------------- 808
MeTPS11   789 DDR-SDEDMFEVITSSMAGPSIAPR----------------------------------- 812
MeTPS12   789 DDR-SDEDMFEVITSSMAGPSIAPR----------------------------------- 812

MeTPS1    844 RSLSTLEG--NTAGSAGWRPIVSDRVSEHEGSSILDLKRENYFSCAVSRKQSIARYLLGT 901
MeTPS2    840 RSLSTLEG--NYLGSGGWRPMVDDRISVHEGSSVLDLKGENYFSCAVSRKRSNARYLLGT 897
MeTPS3    857 RPSPNPDKKLNNHSCGSGRRQSPEKISWN----VLDLKGDNYFSCSVGRTRTNARYLLHS 912
MeTPS4    855 RPSPNLDKKMTNNSCASGRRQSPEKISWN----VLDLKGDNYFSCAVGRTRTNARYLLQS 910
MeTPS5    812 ---------------------------------------PEIFACTVGQKPSKAKYYLDD 832
MeTPS6    808 ---------------------------------------PEIFACTVGRKPSKAKYYLDD 828
MeTPS7    808 ---------------------------------------PEIFACTVGQKPSKARYYLDD 828
MeTPS8    808 ---------------------------------------PEIFACTVGQKPSKARYYLDD 828
MeTPS9    808 ---------------------------------------TSVFACTVGQKPSKAKYYLDD 828
MeTPS10   809 ---------------------------------------AEVFACTVGQKPSKAKYYLED 829
MeTPS11   813 ---------------------------------------AEVFACTVGRKPSKAKYYLDD 833
MeTPS12   813 ---------------------------------------AEVFACTVGRKPSKAKYYLDD 833

MeTPS1    902 SDDVVALLKQMADSSSLT                 919
MeTPS2    898 TDDVVTLLKQLADCLH                   913
MeTPS3    913 SDDVVSFLKKLARASS                   928
MeTPS4    911 SDDVVSFLKKLANASP                   926
MeTPS5    833 AVDVVKLLQGLATASCPKPKHTEQVLVSFESAI  865
MeTPS6    829 TVDVVKLLQGLSAASCPKPKCIENILVSFESAI  861
MeTPS7    829 TVDVLALLQGLANASSSKLRVSTEVQVSFDNLV  861
MeTPS8    829 TVDVLALLQGLAAASSSKPRGSEEVQVSFDNVI  861
MeTPS9    829 TTEVINMLEALAEASDSSPSPGNSP          853
MeTPS10   830 TSEILRMLEGLANASELAARSSPPIGAKTDH    860
MeTPS11   834 TAEIVRLMQGLASVSEQTVNNVS            856
MeTPS12   834 TTEIVRLMQGLASVSEQTVAV              854
```

Figure S1. Predicted enzymatic domains and activity sites in cassava TPS proteins. Predicted TPS domains are highlighted in red; Predicted TPP domains are highlighted in green; Conserved motifs are highlighted in bold. GG1–4: Binding sites 1–4 to Glucose moiety in Glucose-6-Phosphate (G6P); GP1–4: binding. sites 1–4 to Phosphate moiety in G6P; UU: binding sites to Uracil moiety in UDPG; UR1–2: binding sites 1–2 to Ribose moiety in UDPG; UP1–5: binding sites 1–5 to phosphate moiety in UDPG; UG1–5: binding sites 1–5 to glucose moiety in UDPG.

```
MeTPP1      1 MDMKSNKSSPVLTDPAPINKSRLGIHSNLLPYPQSGASFSSGKHINIPRKKPGKL----D  56
MeTPP2      1 MDINSNNSSPVLTDPAPIN---------LLPYPQSGASFSS----NIPRKKPEKL----D  43
MeTPP3      1 MDLKSNHTAPVLTDPAPISKSRLGVHSSLLPYSP-GTAFSSNLFLTIPWKKTGVL----D  55
MeTPP4      1 MDLKSNHTAPVLTDPAPISKSRMGVHSSLLPYTP-GAAFSSNLFLTIPRKKTGVL----D  55
MeTPP5      1 MTN-QNVVVSDPKSTINLAITVHVSDSSIFPTAAQKPPAAPGGYISISRKKLLKNLEING  59
MeTPP6      1 MTN-QNVVVSDPKTTINLATTVHVSNSSIFPTAAQKPPAAPGGYISISRKKLLNHLEINE  59
MeTPP7      1 MTN-QNVVASDAKSGIDIRIRVILP-KSLFSSAEPKPLPAPR-----LAKKIETG---TA  50
MeTPP8      1 MTN-QNVVASDEKSAVDIRIRFLLP-KSLFSPDGPKPLPAPR-----LAKKIETGGVATA  53
MeTPP9      1 MFT-RN--IAKFNQAMGFQRS-----STNKPKTQPGSGRSIR------------------  34
MeTPP10     1 MF-KRS--IAKLNQAMGFQRS-----STNKPKMQPGNDRSTS------------------  34

MeTPP1     57 DVRSNGWLDAMKSSSPPRKKLIKDFNFEVAADET--DIAYFSWMLKYPSALNSFEQITNF 114
MeTPP2     44 EVHYNGWLDAMKSSSPPRKKLIKDLNFEDAADEIEIEIAYFSWMLKYPSALNSFGQITKF 103
MeTPP3     56 DVRSSSWLDAMKSSSPTHKRITKEN----ASADN--DVAYATWTLKYPSAIASFEQIANF 109
MeTPP4     56 DFRSSSWVDTMKSSSPPHKKMTKDLSNEISFADT--DVAYRTWTLKYPSALTSFEQIANF 113
MeTPP5     60 GARINAWVDSMRASSPTHLKSTPSITD-----------DQGSWILHHPSALDMFEQIIEA 108
MeTPP6     60 VARINAWVDSMRASSPTHIKSTPSITD-----------DQGSWILRQPSALDMFEQIIDA 108
MeTPP7     51 AAKTNAWVDSMRDSSPTRVKSTASLSET---------EEKNSWIMNHPSALSMFEQIVNA 101
MeTPP8     54 AAKTNAWVNSMRDSSPTRVKSTCSLSES---------EEKNSWIMNHPSALSMFEQIVNA 104
MeTPP9     35 -MGASNLCHH-IAGGPISIED----------------ASYISWVVEHPSALRSFDQMMKA  76
MeTPP10    35 -MRTTNIKSHPITSGPISIND----------------ASYDTWVVEHPSALGSFDQMMKA  77

                         Motif I                          Motif II
MeTPP1    115 AKTKKIAIFLDYDGTLSPIVVDPDQATMSDEMRSVVRNVAKYFPTAIISGRSRDKVYELV 174
MeTPP2    104 AKNKKIAIFLDYDGTLSPIVDDPDQALMSDDMRSAVRNVAKYFPTAIITGRNRDKVFELV 163
MeTPP3    110 AKGKRIALFLDYDGTLSPIVDNPDRAFMSDAMRSAVEKVAKCFPTAIISGRSRDKVHEFV 169
MeTPP4    114 AKGKRIALFLDYDGTLSPIVDNPDCAFMSNAMRSAVKKVAKCFPTAIISGRSRDKVYEFV 173
MeTPP5    109 SKGKQIVMFLDYDGTLSPIVDDPDRAFMSKKMRATVRKLAKCFPTAIVSGRCRDKVYNFV 168
MeTPP6    109 SKGKQIVMFLDYDGTLSPIVDDPDRAFMSKKMRATVRKLARCCPTAIVSGRCRDKVYNFV 168
MeTPP7    102 SKGKQIVMFLDYDGTLSPIVEDPDRAFMTNEMREAVRDVARYFPTAIVTGRCRDKIYSFV 161
MeTPP8    105 SKGKQIVMFLDYDGTLSPIVEDPDRAFMTNEMREAVRDVARYFPTAIVTGRCRDKVYSFV 164
MeTPP9     77 AKGKKIALFLDYDGTLAPIVDNPDLAFMSDEMRAAVREVAKYFPTAIISGRSRDKVKEFV 136
MeTPP10    78 AKGKKIAVFLDYDGTLAPIVDNPDLAFMSDEMRSAVREVAKYFPTFIITGRSRDKVKEFV 137

MeTPP1    175 GLTELYYAGSHGMDIMGPINKAVSNNQPNCIKSIDQQGKEVNLFQPAREFIPMIDEVFRT 234
MeTPP2    164 GLTELYYAGSHGMDIMGPVNEAVSSNHPDCIKSTDQQGKEVNLLQPARDFIPMIDEVFTT 223
MeTPP3    170 GLKELYYAGSHGMDIMGPVRQYISDDQPNSVRSTDEQGKEVNLFQPASEFLPMIDEVYSS 229
MeTPP4    174 GLTELYYAGSHGMDIMGPVRQSVSDDQPNCMRSADKQGKEVNLFQPASEFLPMIDEVYSS 233
MeTPP5    169 RLAELYYAGSHGMDIKGPA-----------KGSKYKKGSEALIFQPASEFLPMIDEVYKE 217
MeTPP6    169 RLAELYYAGSHGMDIKGPA-----------KGSKYKKESEALIFQPASEFLPMIDEVYKE 217
MeTPP7    162 KLAGLYYAGSHGMDIKGPS-----------KSRKYKKGHQALLFQPASKFLPMIDEVYKV 210
MeTPP8    165 KLAGLYYAGSHGMDIKGPS-----------KSRKYKKDQQALLFQPASKFLPMIDEVCKV 213
MeTPP9    137 QLSNVYYAGSHGMDIMAPPRPVKTCDGKYPTVALDKKGNEV-LFQPAKKFLPAIQKIQTA 195
MeTPP10   138 QLSNVYYAGSHGMDILAPPRPVKSCDGKYQSIALDKKGNEV-LFQPAKKFLPAIQKIQTA 196
```

Figure S2. *Cont.*

MeTPP1	235	LVENTKEIKGAKVENHKFCASVHYRNVDEKNWHTIAQCVHDILTQYPRLRLTHGRKVLEI	294
MeTPP2	224	LVENTKDIKGARVENHKFCASVHYRNVDEKNWPIIAQRVHDVLKLYPRLRLAHGRKVLEV	283
MeTPP3	230	LVENTKYIKGVKVENNKFCVSVHYRNVDDKSWKSVAQCVYDVIKNYPRLRLTHGRMVLEV	289
MeTPP4	234	LVDSTKDIKGAKVENNKFCVSVHYRNVDEKSWKSVAQCVYDVIKNYPRLRLTHGRKVLEV	293
MeTPP5	218	LIEKTKSTPGAKVENNKFCVSVHFRCVDEKKWSELAQVVRSVLKDYPKLRLTQGRKVLEI	277
MeTPP6	218	LVEKTKSTPGAKVENNKFCLSVHFRCVDEKKWSELGQVVRSVLKDYPKLRLTQGRKVLEI	277
MeTPP7	211	LIEKTKSISGAKVENNKFCVSVHFRCVEEKMWAALAEQVRSVLNDYPKLRLTQGRKVLEI	270
MeTPP8	214	LVEKTKSIPGAKVENNKFCVSVHFRCVEEKMWAALAEQVRSALNDYPQLRLTQGRKVLEI	273
MeTPP9	196	LKEKVVEIQGAMVEDNSFCVSVHFRQVREKDYGILEKQVKSVLEHYPEFHLSWGKKVMEI	255
MeTPP10	197	LEEKVVKIQGARIEDNRFCISVHFRQVREEDYETLEKDVKSVLEHYPEFHLSWGKKVMEI	256

Motif III

MeTPP1	295	RPVIDWN**K**GRAVEFLLESLGLSKRDDVLPIYI**GD**DRT**D**EDAFKVLRERNQGYGILVSCVP	354
MeTPP2	284	RPEIDWN**K**GKAVEFLLESLGLGNSDDVLPIYI**GD**DRS**D**EDAFKVLREGNRGYGILVSSAP	343
MeTPP3	290	RPVINWN**K**GKAVTFLLESLGLSNCDDVLPIYV**GD**DRT**D**EDAFKVLRERNCGLGILVSPVP	349
MeTPP4	294	RPVINWD**K**GKAVTFLLESLGLSNCDDVLPIYV**GD**DRT**D**EDAFKVLRERNCGYGVLVTSVP	353
MeTPP5	278	RPTIKWD**K**GKALEFLLESLGFANCTDVFPVYI**GD**DRT**D**EDAFKVLRERGQGFGILVSKFP	337
MeTPP6	278	RPTIKWD**K**GKALEFLLEYLGFTNCTDVFPVYI**GD**DQT**D**EDAFKVLRERGQGFGILVSGIP	337
MeTPP7	271	RPTIKWD**K**GKALEFLLESLGYANSNDVLPVYI**GD**DRT**D**EDAFKVLRNRGQGLGILVSKFP	330
MeTPP8	274	RPTIKWD**K**GKALEFLLESLGYANSNDVLPVYI**GD**DRT**D**EDAFKVLRNRGQGLGILVSKFP	333
MeTPP9	256	RPSIEWD**K**GHALEYLLDTLGLSNSTDVLPLYI**GD**DRT**D**EDAFKVIQRRGQGYPIIVSSSP	315
MeTPP10	257	RPSIQWD**K**GDALEYLLDTLGLSNSNDVIPVYI**GD**DRT**D**EDAFKVIQRRGQGYPIIVSSSP	316

MeTPP1	355	KETNAFYSLRDPIEVLQFLSSLVRWKKLGEER-RSMNNRRSI	395
MeTPP2	344	KETNAFYSLRDPSEVMKFLCWLVRWKKLGEGV-SSVNKRWSI	384
MeTPP3	350	KETNAFYSLRDPSEVMEFLEYLVMWKK	376
MeTPP4	354	KESNAFYSLRDPSEVMEFLKSLVMWKKSSAL	384
MeTPP5	338	KDTNASYSLQEPTQVMDFLQRLVEWKQVSLRG-QPRL	373
MeTPP6	338	KDTNASYYLQEPTQ	351
MeTPP7	331	KETNASYSLQEPTEVKDFLRRLVEWKRFSIG	361
MeTPP8	334	KETNASYSLQEPAEVKDFLRRLVEWKRISFAR-ACRV	369
MeTPP9	316	KDTKASFSLHDPSEVLTFLSRLARWRKSSSSS-RSLAQIWGVGD	358
MeTPP10	317	KDTKASFSLHDPSEVLTFLLRLARWRKSSSSSSRSLAQIWGVSN	360

Figure S2. Predicted enzymatic domains and active motifs in cassava TPP proteins. Predicted TPP domains are highlighted in green; Conserved motifs are highlighted in bold.

Table S1. Accession numbers of genes used in this study.

**Cassava**		**Maize**	
**Gene Name**	**Accession Number**	**Gene Name**	**Accession Number**
MeTPS1	cassava4.1_001215m.g	*ZmTPSI.1.1*	GRMZM2G068943_T01
MeTPS2	cassava4.1_001223m.g	*ZmTPSI.1.2*	GRMZM2G001304_T01
MeTPS3	cassava4.1_021281m.g	*ZmTPSII.2.1*	GRMZM2G019183_T02
MeTPS4	cassava4.1_023440m.g	*ZmTPSII.2.2*	GRMZM2G099860_T01
MeTPS5	cassava4.1_001517m.g	*ZmTPSII.3.1*	GRMZM2G304274_T01
MeTPS6	cassava4.1_001537m.g	*ZmTPSII.3.2*	GRMZM2G123277_T01
MeTPS7	cassava4.1_001541m.g	*ZmTPSII.3.3*	GRMZM2G118462_T01
MeTPS8	cassava4.1_029065m.g	*ZmTPSII.4.1*	GRMZM2G527891_T01
MeTPS9	cassava4.1_001591m.g	*ZmTPSII.4.2*	GRMZM2G008226_T01
MeTPS10	cassava4.1_001558m.g	*ZmTPSII.4.3*	GRMZM2G366659_T01
MeTPS11	cassava4.1_001575m.g	*ZmTPSII.5.1*	GRMZM2G007736_T02
MeTPS12	cassava4.1_001584m.g	*ZmTPSII.5.2*	GRMZM2G079928_T01
MeTPP1	cassava4.1_009204m.g	*ZmTPSII.5.3*	GRMZM2G312521_T01
MeTPP2	cassava4.1_031249m.g	*ZmTPSII.5.4*	GRMZM2G122231_T01
MeTPP3	cassava4.1_009837m.g	*ZmTPPA.1*	GRMZM2G178546_T01
MeTPP4	cassava4.1_031945m.g	*ZmTPPA.3*	GRMZM2G112830_T01
MeTPP5	cassava4.1_009931m.g	*ZmTPPB.1.1*	GRMZM2G347280_T01
MeTPP6	cassava4.1_026633m.g	*ZmTPPB.1.2*	GRMZM2G140078_T01
MeTPP7	cassava4.1_028041m.g	*ZmTPPB.1.3*	GRMZM2G174396_T01
MeTPP8	cassava4.1_010043m.g	*ZmTPPB.1.4*	GRMZM2G055150_T01
MeTPP9	cassava4.1_022149m.g	*ZmTPPB.1.5*	GRMZM2G151044_T01
MeTPP10	cassava4.1_032922m.g	*ZmTPPB.1.6*	GRMZM2G080354_T01
MeTRE1	cassava4.1_004491m.g	*ZmTPPB.2.1*	GRMZM2G014729_T01
MeTRE2	cassava4.1_015916m.g	*ZmTPPB.2.2*	GRMZM2G117564_T01
	cassava4.1_023202m.g	*ZmTPPB.2.3*	GRMZM5G840145_T01
**Rice**		**Arabidopsis**	
**Gene Name**	**Accession Number**	**Gene Name**	**Accession Number**
OsTPS1	OS05G44210	*AtTPS1*	AT1G78580
OsTPS2	OS01G54560	*AtTPS2*	AT1G16980
OsTPS3	OS01G53000	*AtTPS3*	AT1G17000
OsTPS4	OS03G12360	*AtTPS4*	AT4G27550
OsTPS5	OS02G54820	*AtTPS5*	AT4G17770
OsTPS6	OS05G44100	*AtTPS6*	AT1G68020
OsTPS7	OS08G31980	*AtTPS7*	AT1G06410
OsTPS8	OS08G34580	*AtTPS8*	AT1G70290
OsTPS9	OS09G25890	*AtTPS9*	AT1G23870
OsTPS10	OS09G23350	*AtTPS10*	AT1G60140
OsTPS11	OS09G20990	*AtTPS11*	AT2G18700
OsTPP1	OS02G44230	*AtTPPA*	AT5G51460
OsTPP2	OS10G40550	*AtTPPB*	AT1G78090
OsTPP3	OS07G43160	*AtTPPC*	AT1G22210
OsTPP4	OS02G51680	*AtTPPD*	AT1G35910
OsTPP5	OS04G46760	*AtTPPE*	AT2G22190
OsTPP6	OS08G31630	*AtTPPF*	AT4G12430
OsTPP7	OS09G20390	*AtTPPG*	AT4G22590
OsTPP8	OS06G11840	*AtTPPH*	AT4G39770
OsTPP9	OS03G26910	*AtTPPI*	AT5G10100
OsTPP10	OS07G30160	*AtTPPJ*	AT5G65140
OsTPP11	OS02G44235	-	-
OsTPP12	OsI_34594	-	-
OsTPP13	OS12G09060	-	-
